# Simplified vs extended in vitro methods for the evaluation of bioaccessibility of metals and metalloids present in urban recreational soils

**DOI:** 10.1007/s11356-025-36017-y

**Published:** 2025-02-09

**Authors:** Ainhoa Lekuona-Orkaizagirre, Maite Meaurio, Ainara Gredilla

**Affiliations:** 1 Department of Applied Chemistry, Faculty of Chemistry, University of the Basque Country (UPV/EHU), Donostia-San Sebastian, Spain; 2 Department of Analytical Chemistry, Faculty of Science and Technology, University of the Basque Country (UPV/EHU), Leioa, Spain

**Keywords:** Soils, Metals and metalloids, Bioaccessibility, SBET, RIVM

## Abstract

Urban soils may contain different metals and metalloids and they can enter the human body by direct inhalation, ingestion or dermic absorption. According to USEPA, 200 mg·day^−1^ is the average daily ingested dose of soil for children aged from 1 to 12 years of age. In vitro bioaccessibility tests which are based on human physiology, have been used for the determination of the element fraction in soils that can be absorbed by human digestion. A total of 26 urban soils were collected in recreational areas from San Sebastian to evaluate the bioaccessibility of metals and metalloids (Al, Ti, V, Cr, Mn, Fe, Co, Ni, Cu, Zn, As, Cd and Pb) by SBET (Simplified Bioaccessibility Extraction Test) and RIVM (Dutch National Institute for Public Health and Environment) in vitro methodologies. SBET simulates the gastric conditions, while RIVM simulates mouth, gastric and intestinal conditions. ICP-MS was used for the simultaneous determination of the elements. Cd showed the highest percentage of bioaccessibility by both methods and Fe, Al and Cr resulted the least bioaccessible elements. Pb and Zn showed different results in each method. HQ (Hazard Quotient), HI (Hazard Index) and CR (Carcinogenic Risk) values obtained were higher with SBET methodology. According to HI (considering 10 metal(loid)s), non-carcinogenic effects may occur to children with the ingestion of three of the studied children’s parks. Regarding CR index, all the studied soils were within the tolerable carcinogenic risk (considering Cr, As and Pb) for children, and the risk was sometimes negligible for adults. Considering the experimental difficulties related to RIVM, SBET method may be used for a simple and conservative first approach of the bioaccessibility of metals and metalloids accumulated in soil samples.

**Supplementary information:**

The online version contains supplementary material available at 10.1007/s11356-025-36017-y.

## Introduction

Soil is defined as the surface layer of the Earth’s crust and is composed primarily of mineral particles, organic matter, water, air and living organisms. The soil is very dynamic and has many functions: it is the main producer of biomass, allows the development of biodiversity and the survival of the ecosystem and it is the main producer of carbon. Soil is fundamental to human beings, as it is the main food producer (Petruzzelli et al. 2020). It is important, therefore, to ensure soil quality.

Urban soil was initially defined as non-agricultural soil material that has been produced by filling land surface in urban areas (Craul 1992). Later definitions use the term “anthropogenic soil” which defines urban soils as human-altered soils (Pouyat et al. 2020). In this context, urban soils, considered as soils located in urban areas that have been affected by human activities, can contain elements of different toxicity that can be found at a concentration below 100 mg·kg^−1^, which are called trace elements. Most inorganic trace elements significant for the environment, humans or animals are metals such as cadmium (Cd), chromium (Cr), cobalt (Co), copper (Cu), gold (Au), lead (Pb), manganese (Mn), mercury (Hg), molybdenum (Mo), nickel (Ni), palladium (Pd), silver (Ag), thallium (Tl), tin (Sn), vanadium (V), platinum (Pt), rhodium (Rh), and zinc (Zn). Other important trace elements are metalloids (boron (B), arsenic (As) and antimony (Sb)), some non-metals (selenium (Se)), actinides (uranium (U)) and a few halogens (iodine (I) and fluorine (F)) (Hooda 2010). Although some metal(loid)s are necessary for life and are called essential elements which are required for biochemical and physiological functions, they can be toxic in large amounts (Briffa et al. 2020).

The soil, together with its (trace) elements, can be incorporated into the human body by direct inhalation, ingestion and absorption by dermal contact (Kharazi et al. 2021; Peng et al. 2022). According to some studies (Du et al. 2013; Różański et al. 2021), the ingestion of soil particles is the predominant pathway to introduce heavy metals into the body, for both children and adults. Some chemicals (including metals and metalloids) present on contaminated surfaces or soils can be transferred to hand and then ingested through hand-mouth activity. The frequency of hand-mouth behaviour, therefore, should be considered to estimate the mass of soil ingested by humans. This path is particularly important for children because as part of their natural development, children tend to take their fingers or other objects into their mouths (Xue et al. 2007). According to the United States Environmental Protection Agency (USEPA), the average daily ingestion values for soils are 200 mg·day^−1^ for children from 1 to 12 years of age and 100 mg·day^−1^ for people over 12 years of age (USEPA 2011). As age increases, the tendency to ingestion decreases, so children are especially sensitive and susceptible to soil intake, and to the consequent toxicological effects of the contaminants present (Xue et al. 2007). When the soil enters the stomach, mineral oxides, sulphides and carbonates, among others, release metals and metalloids (Ljung et al. 2007). Then, in the intestinal phase, the higher pH of the intestines dissolves organic matter, and, consequently, contaminants bound to them are released. Some of these released metals are solubilised by complexation with bile acids and others are precipitated together with phosphates (Grøn and Andersen 2003; Ljung et al. 2007). After being transported through the intestinal wall, metals can be transferred to the bloodstream (Oomen et al. 2002). Over time, metal(loid)s can be bioaccumulated in our system and they can cause biological and physiological complications. Cellular components can be affected by metal(loid)s, such as the mitochondria, nuclei, lysosomes, cell membrane and enzymes (Briffa et al. 2020). Metal(loid) ions interact with DNA and nuclear proteins, causing DNA damage, leading to cell cycle modulation, apoptosis or carcinogenesis (Briffa et al. 2020). In addition, it has been reported that metal(loid) toxicity can damage the functioning of the brain, lungs, kidney, liver and blood composition. Long-term exposure may lead to progressive physical, muscular, and neurological degenerative processes. Repeated long-term exposure of some metals may even cause cancer (Järup 2003). Some studies have investigated the relationship between metal contents in soils and blood metal contents in children, and concluded that dust inhalation or ingestion can cause an elevation of blood lead level (BLL) (He et al. 2020). Children are particularly susceptible to lead exposure due to the high gastrointestinal uptake and also due to their permeable blood–brain barrier (Järup 2003).

Considering the above, for the evaluation of the effect of metals and metalloids coming from the ingestion of soil, especially in the case of children, two terms can be cited: oral bioavailability and bioaccessibility. According to Oomen et al. (2002) the oral bioavailability of soil contaminants is defined as the contaminant fraction that reaches the systemic circulation, whereas the bioaccessible fraction is defined as the fraction of the contaminant that is mobilised from soil into the digestive juice chyme. In vivo experiments with animals anatomically, metabolically and physiologically similar to humans can be performed to evaluate bioavailability (Turner 2011). As a more ethical and economical alternative, it is possible to calculate through in vitro tests the soluble fraction of a contaminant in the gastrointestinal tract and, thus, the bioaccessible fraction. According to Oomen et al. (2003), an in vitro methodology should meet two requirements: on the one hand, it should be based on human physiology and especially take into account child physiology, due to their hand-to-mouth behaviour and so their higher vulnerability. On the other hand, it should represent the worst-case situation, always as realistic as possible. In other words, high priority elements should be chosen, and when a physiological value (such as gastric pH) varies in each individual, in order to simulate the worst-case scenario, it should be chosen the value where this element mobilizes more and gives greater bioaccessibility value (Oomen et al. 2003). For instance, a literature review indicated that the gastric pH value in children varies from 1 to 4 (Anderson et al. 1999), and it has been reported that lead bioaccessibility decrease with increasing gastric pH (Oomen et al. 2003). Oomen and his colleagues (2003), therefore, chose the pH 1 value to develop their bioaccessibility test, as is was thought to be the worst-case situation for lead, thus giving higher bioaccessibility values. Among the in vitro methodologies for the quantification of the bioaccessible contaminant fraction in soils, the following can be remarked: SBET (Simplified Bioaccessibility Extraction Test) simulates human gastric conditions; PBET (Physiologically Based Extraction Test) or IVG (In Vitro Gastrointestinal Environment Method) take into account gastric and intestinal phases; and RIVM (Dutch National Institute for Public Health and Environment) that simulates mouth, gastric and intestinal conditions.

The main objectives of this study were: (1) to determine the bioaccessibility of the greatest possible number of metals and metalloids using different in vitro techniques, (2) to characterize non-carcinogenic and carcinogenic human health risks associated to the ingestion of soils collected in urban parks and (3) to compare the results obtained from two in vitro methods. RIVM and SBET methods were used in this case for the determination of bioaccessibility, the first simulated the whole human digestion and the latter simulated only the gastric phase, making it simpler and faster. The health risks were evaluated with the calculation of HQ (Hazard Quotient) and HI (Hazard Index) for non-carcinogenic risk and CR (Carcinogenic Risk) index.

## Materials and methods

### Sampling and sample preparation

**Fig. 1:**
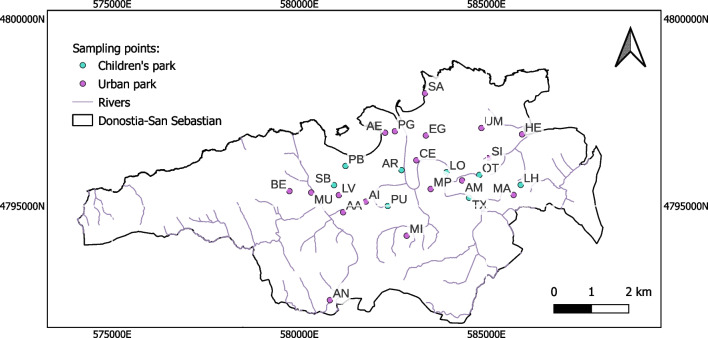
Location and identification of urban parks sampled in Donostia-San Sebastian

A total of 26 urban soil samples were collected in some parks in Donostia-San Sebastian (43.3128º N, 1.975º W), a city located in the Basque Country, in northern Spain and near the border with France, close to the Cantabrian coast. The city presents, due to its proximity to the Cantabrian Sea, a temperate oceanic climate, characterized by mild temperatures, relatively high humidity, frequent cloudiness and annual precipitations which exceed 1500 mm, distributed regularly throughout the year (Gómez-Piñeiro, 1999). There are 182,000 inhabitants in the city and the economic activity focuses on the services sector, specifically on tourism. This sector can generate a great amount of waste which is degraded and can be a source of metals and metalloids (Eustat 2023). Parks with benches, walking or cycling paths, trees and plants were considered urban parks for this study, and parks with swings or slides were considered children’s parks. Figure 1 shows the location of each sampling site on a map. Composite samples, made up of three subsamples were collected with a stainless steel hoe in each point from the superficial soil layer (0–20 cm depth) and they were transferred into air-tight polyethylene bags for their transport to the laboratory. Once in the laboratory, the soil samples were dried at room temperature for at least 72 h. Then samples were sieved below < 2 mm and stored in polyethylene bags at 4 ºC until analysis.

### Soil analysis

Before the bioaccesibility assessment some physicochemical properties of soils were determined. A soil–water suspension (5:25) was prepared with each sample and for the measurement of pH, the suspension was agitated for 1 h and then centrifuged at 3000 rpm for 10 min. pH was measured with “Basic 20 pH, Crison” pHmeter. For the measurement of electrical conductivity, the suspension was agitated for 30 min and then was left to stand for 15 min for the separation of liquid and solid phases. “4510 Conductivity Meter, JENWAY” was used for the measurement and a KCl 0.010 M solution was prepared for the calibration. Organic matter (OM) was determined by weight Loss On Ignition (LOI) at 550ºC for 4 h (Schumacher 2002).

The mineralogical analysis of soils was performed by X-ray diffraction (XRD) with a PANalytical Xpert PRO diffractometer, equipped with a copper tube, a vertical goniometer (Bragg–Brentano geometry), a programmable divergence slider, an automatic sample exchanger, a secondary monochrome and a PixCel detector. For soil mineralogy, an aliquot portion of each sample was manually ground using an agate mortar, up to a grain size suitable for the analysis of disoriented powder sample. The instrumental conditions used were: a generator current of 40 kV and 40 mA and an angular scanning of 5–70º2q. For the analysis of clay minerals, the samples were crushed without grinding, were decarbonised with diluted HCl (if applicable) and were washed by centrifugation until chlorides were eliminated. Then, the < 2 micron fraction was extracted (Stokes’ Law, centrifugation) and the oriented aggregates (OA) of that fraction were prepared. The identification of the different groups of clay minerals was carried out comparing the diffractograms of the OAs subjected to different treatments: OAst without treatment, OAeg solvatation with ethylene glycol, OAdm solvatation with dimethyl sulfoxide, OAtt heat treatment at 550ºC and OA_300 heat treatment at 300ºC. Diffractograms of the OA were measured and interpreted together. For the OAs, the instrumental conditions used were: a generator current of 40 kV and 40 mA and an angular scanning of 2–30º2θ.

Pseudototal metal and metalloid content of soils was determined following the US Environmental Pollution Agency EPA 3051A method. Thus, 0.5 g of soil (< 2 mm) was weighted and 9 mL HNO_3_/3 mL HCl acid mixture was added. The acid digestion was carried out by keeping the solution at 170ºC for 50 min, in a “speedwave” microwave oven (Berghof). After cooling, the digests were filtered below 0.45 µm and transferred to polyethylene bottles of 50 mL (Gredilla et al. 2017). The concentration of metals and metalloids (Al, Ti, V, Cr, Mn, Fe, Co, Ni, Cu, Zn, As, Cd and Pb) was determined by an Inductively Coupled Plasma—Mass Spectrometer (ICP-MS 7700, Agilent Technologies) using four internal standards (Sc, Ge, Rh and Ir) to assess the stability of ICP-MS measures. Quality controls consisted of various reagents blank for the determination of the Limit of Detection (LOD), three triplicate samples for the determination of the Relative Standard Deviation (RSD) and a standard reference material (NIST 1646a) (National Bureau of Standards Certificate of Analysis) for the determination of recoveries.

### Determination of in vitro bioaccessibility

#### Experimental procedure

RIVM in vitro digestion was carried out as described by Oomen et al. (2003). As this method simulates the three phases in the human digestion (mouth processing, gastric processing and intestinal processing), three synthetic solutions were prepared with the aim of reproducing saliva, gastric juice and intestinal juices (duodenal juice and bile) (Table S1). The inorganic solution, organic solution and additional substances were mixed and MilliQ water was included to obtain 500 mL of each synthetic solution.

The digestion started introducing 9 mL of saliva to 0.6 g of soil (< 2 mm). This mixture was agitated for 5 min at 37ºC. Then, 13.5 mL of gastric juice were added and the mixture was agitated for 2 h. Finally, 27 mL of duodenal juice and 9 mL bile were added simultaneously and the mixture was agitated for 2 h. Temperature was maintained at 37ºC in the whole digestion procedure. Then, the solution was filtered below 0.45 µm and the metals and metalloids (Al, Ti, V, Cr, Mn, Fe, Co, Ni, Cu, Zn, As, Cd and Pb) in the filtrate were analysed by ICP-MS (Oomen et al. 2003).

SBET is a simplified in vitro digestion method which simulates the gastric phase. The synthetic stomach fluid was prepared by dissolving 60.06 g glycine in 2 L of MilliQ water and adjusting it to pH 1.5 with HCl (12.1 M). The digestion was simulated by adding 50 mL of the artificial stomach fluid to 0.5 g of soil sample (< 2 mm) and the mixture was agitated for 1 h at 37ºC. Then, the solution was filtered below 0.45 µm and the metals and metalloids (Al, Ti, V, Cr, Mn, Fe, Co, Ni, Cu, Zn, As, Cd and Pb) in the filtrate were analysed by ICP-MS (Ning et al. 2021; Kim et al. 2002).

#### Calculation of bioaccessibility (%)

SBET and RIVM bioaccessibility of each metal or metalloid obtained for each sample was calculated by Eq. 1 and the average bioaccessibility value was obtained for each metal or metalloid considering the values obtained from the 26 samples.

1 $${Bioaccessibility}_{SBET/RIVM} \left(\mathrm{\%}\right)=\frac{{\text{Element bioaccessible concentration }\left(\frac{\mathrm{mg}}{\mathrm{kg}}\right)}_{SBET/RIVM}}{\text{Element pseudototal concentration }\left(\frac{\mathrm{mg}}{\mathrm{kg}}\right)}\cdot 100$$

#### Quality control of the methods

Several blank reagents were prepared and measured for each method (pseudototal, SBET, RIVM), using the same procedure but without adding the soil. Then, the Limit Of Detection (LOD) of each method, thus, the minimum concentration detected, was calculated by Eq. 2 (SD: standard deviation of the blank signal and a: calibration slope for each metal/metalloid). To estimate the magnitude of the blanks of SBET and RIVM with respect to the average concentration of each element, an average blank concentration was calculated for each metal/metalloid and then the contribution of each blank was estimated considering all the samples (Barsby et al. 2012). Finally, the blank percentage (blank %) was calculated by Eq. 3.

2 $${LOD}_{SBET/RIVM}^{metal}=\frac{3.3\cdot {SD}_{SBET/RIVM}^{metal}}{{a}_{SBET/RIVM}^{metal}}$$

3 $${\mathrm{Blank}}_{\mathrm{SBET}/\mathrm{RIVM}}^{\mathrm{\%}}=\frac{{\mathrm{Blank}}_{\mathrm{SBET}/\mathrm{RIVM}}^{\mathrm{metal}}}{{\mathrm{Mean}}_{\mathrm{SBET}/\mathrm{RIVM}}^{\mathrm{metal}}}\cdot 100$$

The repeatability of both methods was studied by measuring three samples in triplicate and calculating their respective RSD (*Relative Standard Deviation*).

### Human health risk assessment

The non-carcinogenic risk associated with the bioaccessibility values obtained was estimated by the Hazard Quotients (HQ). HQ are based on the relative bioaccessibility recommended by USEPA (2007) and are calculated by Eq. 4 (Yu and Yang 2019). HQ ≤ 1 indicates no adverse health effects and HQ > 1 indicates likely adverse health effects (Gu et al. 2016).

4 $$\mathrm{HQ}=\frac{\mathrm{DI }\cdot \mathrm{ RBA}}{\mathrm{RfD}}$$

Relative Bioaccessibility (RBA) was the ratio of metals’ concentrations extracted using SBET or RIVM method to their total concentrations in soils. DI is the daily oral intake (mg·kg^−1^·day^−1^) calculated by Eq. 5 (USEPA 2007) and RfD the reference dose of daily intake (mg·kg^−1^·day^−1^). In the study, the non-carcinogenic risk was calculated for metals and metalloid which have a defined RfD value (mg·kg^−1^·day^−1^): 3.0E-03 for Cr, 2.4E-02 for Mn, 7.0E-01 for Fe, 3.0E-04 for Co, 2.0E-02 for Ni, 4.0E-02 for Cu, 3.0E-01 for Zn, 3.0E-04 for As (USEPA 2022); 5.0E-04 for Cd and 3.5E-03 for Pb (Gu et al. 2016).

5 $$\mathrm{DI}=\frac{\mathrm{C }\cdot \mathrm{ IR }\cdot \mathrm{ EF }\cdot \mathrm{ ED }\cdot \mathrm{ CF}}{\mathrm{BW }\cdot \mathrm{ AT}}$$

C is the total metal/metalloid content (*pseudo*total in this study), mg·kg^−1^; IR the ingestion rate, set as 200 mg·day^−1^ for children (< 12 years) and 100 mg·day^−1^ for adults (> 12 years) (USEPA 2017; Gu et al. 2016); EF the exposure frequency which was set as 350 days·year^−1^ (Gu et al. 2016); ED is exposure duration, in this study 6 years for children and 30 years for adults (Gu et al. 2016); CF is the conversion factor of 1·10^–6^ kg·mg^−1^ and AT the averaging time (for non-carcinogens, AT = ED · 365; for carcinogens AT = 70 · 365) (Gu et al. 2016; Yu and Yang 2019). BW is the average body mass: 70 kg for adults (> 18 years) and 12 kg for children (1–3 years) (EFSA 2012).

With the aim of understanding the non-carcinogenic risk of all the above mentioned elements simultaneously, Hazard index (HI) was calculated, which is equal to the sum of HQ of different metals. When HI value is smaller than one, there is no significant risk of non-carcinogenic effects, and if HI exceeds one, there is a chance that non-carcinogenic effects may occur (Gu et al. 2016). In this study, HI was calculated as the sum of the HQ index of 10 metal(loid)s (Cr, Mn, Fe, Co, Ni, Cu, Zn, As, Cd and Pb).

The Carcinogenic Risk (CR) was calculated for metals and metalloids which have a defined CSF value (Cr, As and Pb) (mg·kg^−1^·day^−1^) by Eq. 6, where DI is the daily intake calculated by Eq. 5, RBA is the relative bioaccessibility (the ratio of metals’ concentrations extracted using SBET or RIVM method to their total concentrations in soils) and CSF the cancer slope factor values for each metal or metalloid. CSF value of Cr (VI) (5.0E-01) was used as the total Cr reference value. CSF values for As and Pb were 1.5E + 00 and 8.5E-03, respectively (USEPA 2022). The estimated value of CR is the probability for an individual to develop cancer over a lifetime exposure to carcinogenic hazards and the tolerable risk for regulatory purposes is between 1·10^–6^ and 1·10^–4^. Less than 1 chance in 1,000,000 (1·10^–6^) is negligible, and chances above 1·10^–4^ are sufficiently large that some sort of remediation is required (Gu et al. 2016). In this work the CR value was calculated for the elements As, Cr and Pb, and then, the sum of the CR indices of the three metal(loid)s was indicated as Total CR.

6 $$\mathrm{CR}=\mathrm{DI}\cdot \mathrm{RBA}\cdot \mathrm{CSF}$$

## Results and discussion

### Soil properties

The range of pH values in soil samples varied between 5.70 and 8.18 and organic matter was between 8 and 25%. Quartz (SiO_2_) and phyllosilicates (clay minerals) were the most predominant minerals, ranged between 20 and 79% and 19% and 63%, respectively. In fact, together they compose almost 100% of the mineralogy of the samples. Phyllosilicates were mainly formed by clay minerals such as Illite (KAl_2_Si_3_AlO_10_](OH)_2_), to a lesser extent by Chlorite ((Mg,Fe)_3_(Si,Al)_4_O_10_(OH)_2_·(Mg,Fe)_3_(OH)_6_) and Kaolinite (Al_2_Si_2_O_5_(OH)_4_), and in some specific urban park samples also appeared Vermiculite (Mg, Fe, Al)_3_[(Si,Al)_4_O_10_(OH)_2_) (in MA, AN and MI) and Smectite (a group of hydroxyl aluminosilicates (mainly Na^+^, Ca^+2^, Mg^+2^, Fe^+3^ and Li^+^) (in SA). In general, calcite (CaCO_3_) was below 5%, except for SA, HE and AN urban park samples. Feldspar presence was null in the analysed parks, except for AR, UL and MI. Table S2 presents the soil properties of each analysed soil, as well as the maximum, minimum and mean values of the data set.

**Fig. 2:**
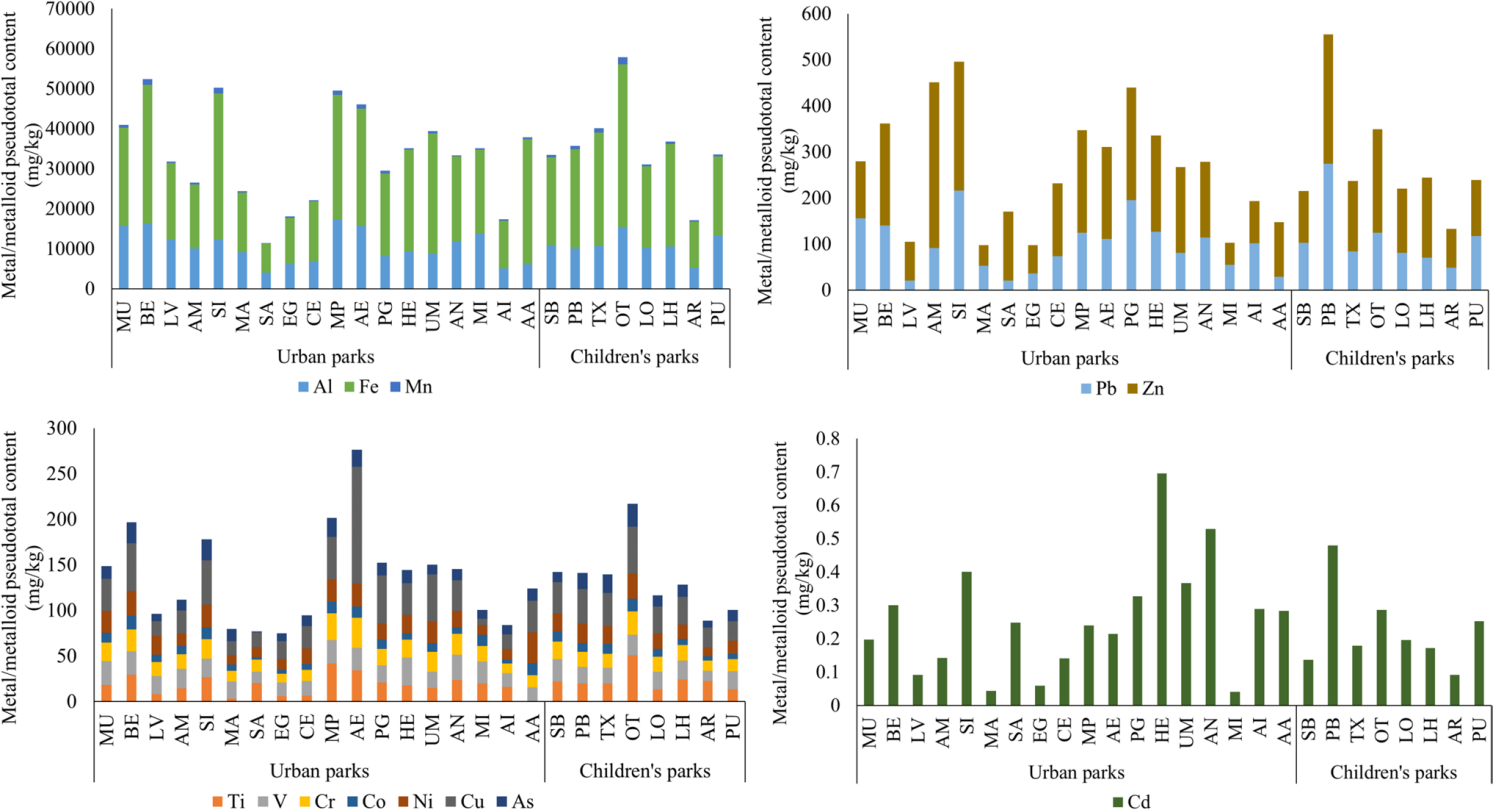
Metal and metalloid pseudototal concentrations (mg·kg^−1^) in the studied 26 soils of San Sebastian

Generally, the order of metal and metalloid concentrations in the urban park soils, from highest to lowest, was the following: Fe > Al > Mn > Zn > Pb > Cu > V > Ti > Ni > Cr > As > Co > Cd (Fig. 2). Fe showed the highest concentrations, with a mean value of nearly 23,000 mg·kg^−1^, and Cd was the scarcest element, with a maximum value lower than 0.70 mg·kg^−1^. RSD values were below 5% for all elements, except for Ti which was 7%. The trueness of the method was confirmed by the analysis of the NIST reference material with satisfactory results (recoveries between 80–100%).

A correlation analysis was done considering all the samples and the soil properties studied (Table S3), and a significant correlation was found between Cd and calcite content (r = 0.645). Samples with highest amount of calcite (HE and AN) were the ones that contained the maximum concentration of Cd (Fig. 2). In fact, calcium carbonate is considered an effective cadmium absorbent, although its absorption mechanism remains unclear (Sasamoto et al. 2022). In contrast, an inverted effect was observed between Cd and quartz content (r = −0.507) (Table S3). Samples with highest amount of quartz (EG and AR) contained low concentrations of Cd (Fig. 2). There was also a significant correlation between the phyllosilicates and Ni content (r = 0.665) (Table S3): AA contained the maximum content of Ni and phyllosilicates (Table S2). Fe and Co content also showed correlation with phyllosilicates. Metal content was generally negatively correlated with Illite content, and positively correlated with Kaolinite or Chlorites (Table S3). For instance, samples with higher amount of Kaolinite (BE, SB, HE) contained higher V concentration (r = 0.506).

VIE-B Indicative Assessment Values defined in Soil Law 4/2015 from the Basque Government are used as reference for the determination of soil contamination. VIE-B values are defined for the protection of people and values are classified depending on the use of the respective soil in five categories: playground for children, city, public park, industry and other uses. VIE-B values for children’s playgrounds, described in the Legislation, are the following: 90 mg·kg^−1^ for Cr, 110 mg·kg^−1^ for Ni, 30 mg·kg^−1^ for As, 5 mg·kg^−1^ for Cd, 120 mg·kg^−1^ for Pb and the derived limit value for Zn and Cu is of the order of tens of g/kg (Basque Government 2015). All pseudototal concentrations obtained were below VIE-B values except for some Pb data. The samples MU, BE, SI, PG, OT, HE and PB exceed the VIE-B limit for playgrounds (Fig. 2). Among the parks exceeding the VIE-B value defined for children’s playgrounds, only PB and OT are dedicated to this use, since they contain slides for children to play.

### Quality control of the methods

As it is reflected in Table 1 the Relative Standard Deviation (RSD%) in the pseudototal and SBET triplicates was below 10% in all elements and RSD values for RIVM triplicates were generally higher because of the complexity of the matrix. On the other hand, the average metal content in the samples after being extracted by SBET method was further from their respective LOD, compared to the RIVM method (Table 1).

**Table 1:** The average metal and metalloid pseudototal, SBET extracted and RIVM extracted concentrations (mg·kg^−1^) together with the Relative Standard Deviation (RSD%) and Limits of Detection (LOD) of each method

	Pseudototal			SBET					
Element	Mean ± SD	RSD_pseudo._%	LOD_pseudo_	Mean ± SD	RSD_SBET_%	LOD_SBET_	Mean ± SD	RSD_RIVM_%	LOD_RIVM_
Al	10,631 ± 254	2.4	211	674 ± 48	7.2	50	11.0 ± 0.8	7.4	0.8
Ti	20 ± 1	7.0	1.6	1.93 ± 0.04	2	0.1	< LOD_RIVM_	13	0.4
V	20 ± 1	4.7	0.62	2.4 ± 0.1	4.4	0.02	0.49 ± 0.04	8	0.22
Cr	17.9 ± 0.5	2.8	1.4	0.7 ± 0.6	9.3	0.1	< LOD_RIVM_	11	0.2
Mn	657 ± 22	3.3	54	335 ± 10	3.1	7	167 ± 23	13	27
Fe	22,855 ± 746	3.3	170	815 ± 79	9.6	29	18 ± 4	20	5
Co	9.1 ± 0.3	3.5	0.6	3.4 ± 0.2	4.8	0.02	2.0 ± 0.1	5.8	0.1
Ni	19.0 ± 0.6	2.9	0.6	2.8 ± 0.5	17	0.2	2.1 ± 0.2	12	0.4
Cu	35.4 ± 0.8	2.4	0.9	13.8 ± 0.6	4.4	0.5	7.9 ± 0.3	4.1	1.6
Zn	164 ± 5	3.0	8.7	61 ± 12	18	18	< LOD_RIVM_	5.5	21
As	14.3 ± 0.8	5.4	2.3	1.47 ± 0.03	1.7	0.002	1.25 ± 0.04	3.1	0.07
Cd	0.246 ± 0.003	1.4	0.011	0.25 ± 0.01	4	0.07	0.104 ± 0.001	1.1	0.07
Pb	102 ± 2	2.1	0.33	84 ± 2	2.8	0.8	0.78 ± 0.07	9.3	0.16

**Fig. 3:**
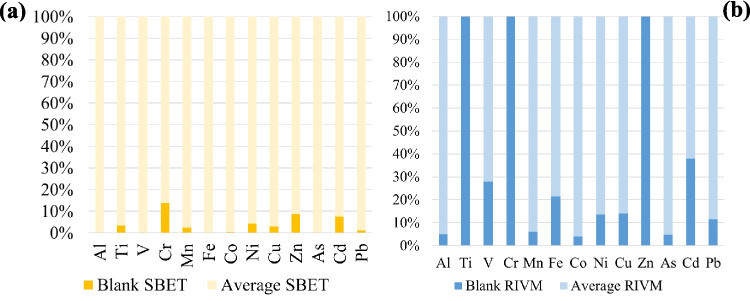
The contribution of the average blank of each element with respect to the average concentration of each element considering all the samples, for SBET (**a**) and RIVM (**b**)

Figure 3 shows the contribution of the average blank of each element with respect to the average concentration of each element, considering all the samples, calculated by Eq. 4, in percentages. The blank percentage values for SBET were always lower than the values obtained for RIVM. For SBET method, all blank extractions for all elements were below 5% of the average concentration of each element, except Cr, Zn and Cd (Fig. 3a). For RIVM method only blank values of Al, Mn, Co and As were < 10% of the average element concentration. The rest fell between 10 and 70% of the average values. Ti, Cr and Zn average values were below the detection limit (Fig. 3b).

The synthetic stomach fluid used in SBET methodology was made up of glycine and HCl, while the synthetic fluids in RIVM formed a complex matrix, composed of organic compounds and inorganic salts, together with enzymes (Table S1). As stated by Barsby et al. (2012), the blank concentrations in gastro-intestinal method tend to be higher due to the addition of biological reagents which are not available in such high purity and thus produce high blanks for some metals.

### SBET and RIVM extractable concentrations and bioaccessibility (%)

SBET and RIVM results can simulate the amount of contaminant absorbed in the human organism by the ingestion of soils. In general, the results showed higher concentration values for all the elements when the SBET method was used instead of RIVM (Table 1). That fact may be related to the acidity of the reagents, since in the SBET method the soil was extracted with a solution of pH 1.5 and in the RIVM method synthetic fluids of variable acidity were added to the soils and digested products of approximately pH 8 were finally obtained. In fact, a precipitation process can occur when moving from acidic (gastric) conditions to alkaline (intestinal) conditions.

**Fig. 4:**
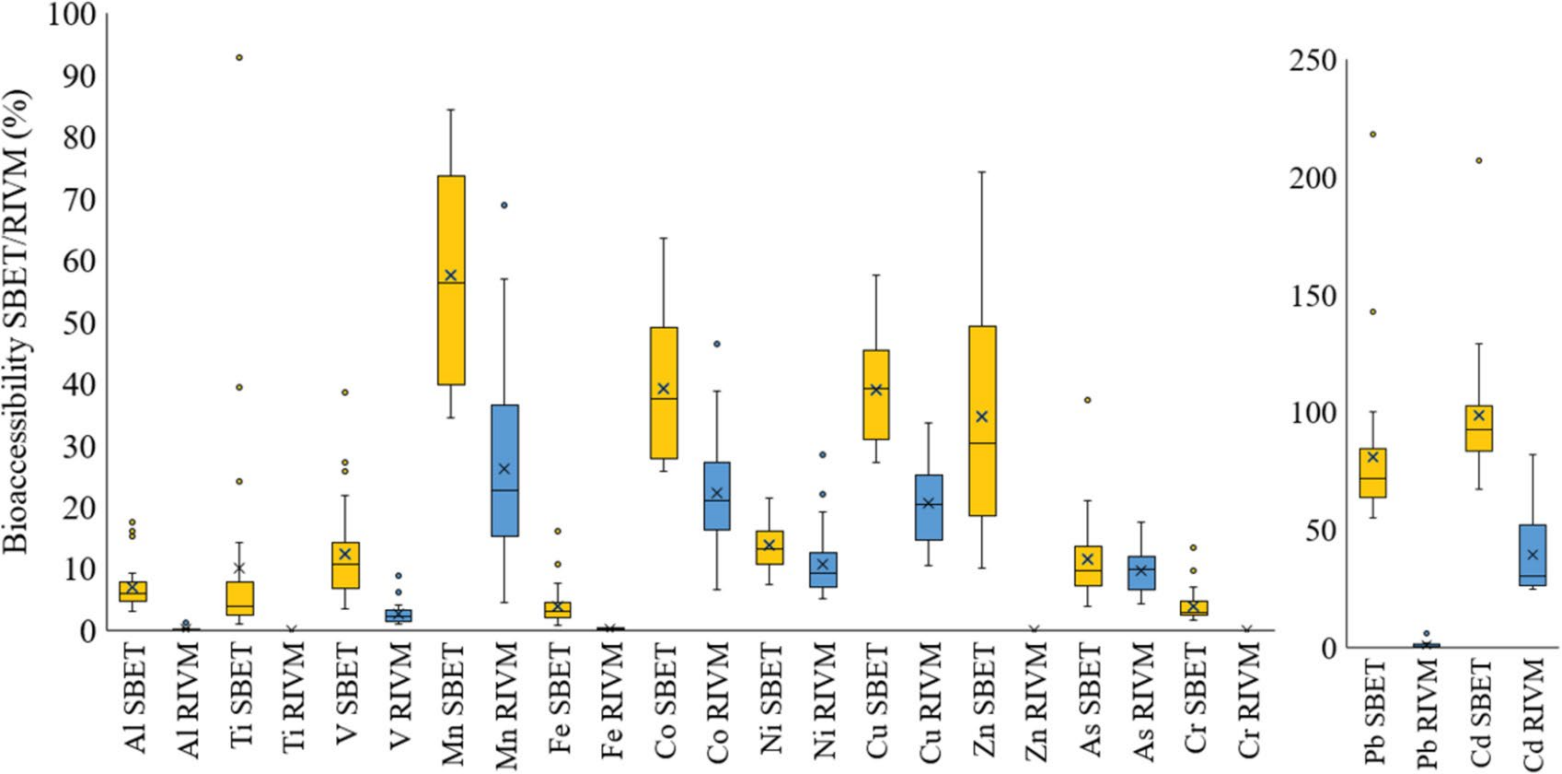
Metal/metalloid SBET and RIVM bioaccessibility (%) in urban park soils. The lines (whiskers) extended from the box indicate the variability outside the upper (75%) and lower (25%) quartiles, the cross (x) indicates de mean value and the individual points represent the outliers that differ significantly from the rest of the dataset

With the aim of assessing the bioaccessibility of each metal/metalloid, Fig. 4 shows the bioaccessibility by SBET and the bioaccessibility by RIVM for each metal/metalloid. Each box represents the dataset of the bioaccessibility percentage (%) of each metal/metalloid in 26 samples, through the quartiles. RIVM bioaccessibility was always lower than SBET bioaccessibility. Similar results were obtained by Roussel et al. (2010), where the gastric bioaccessibility was twice the gastro-intestinal bioaccessibility for Cd, Zn and Pb.

**Fig. 5:**
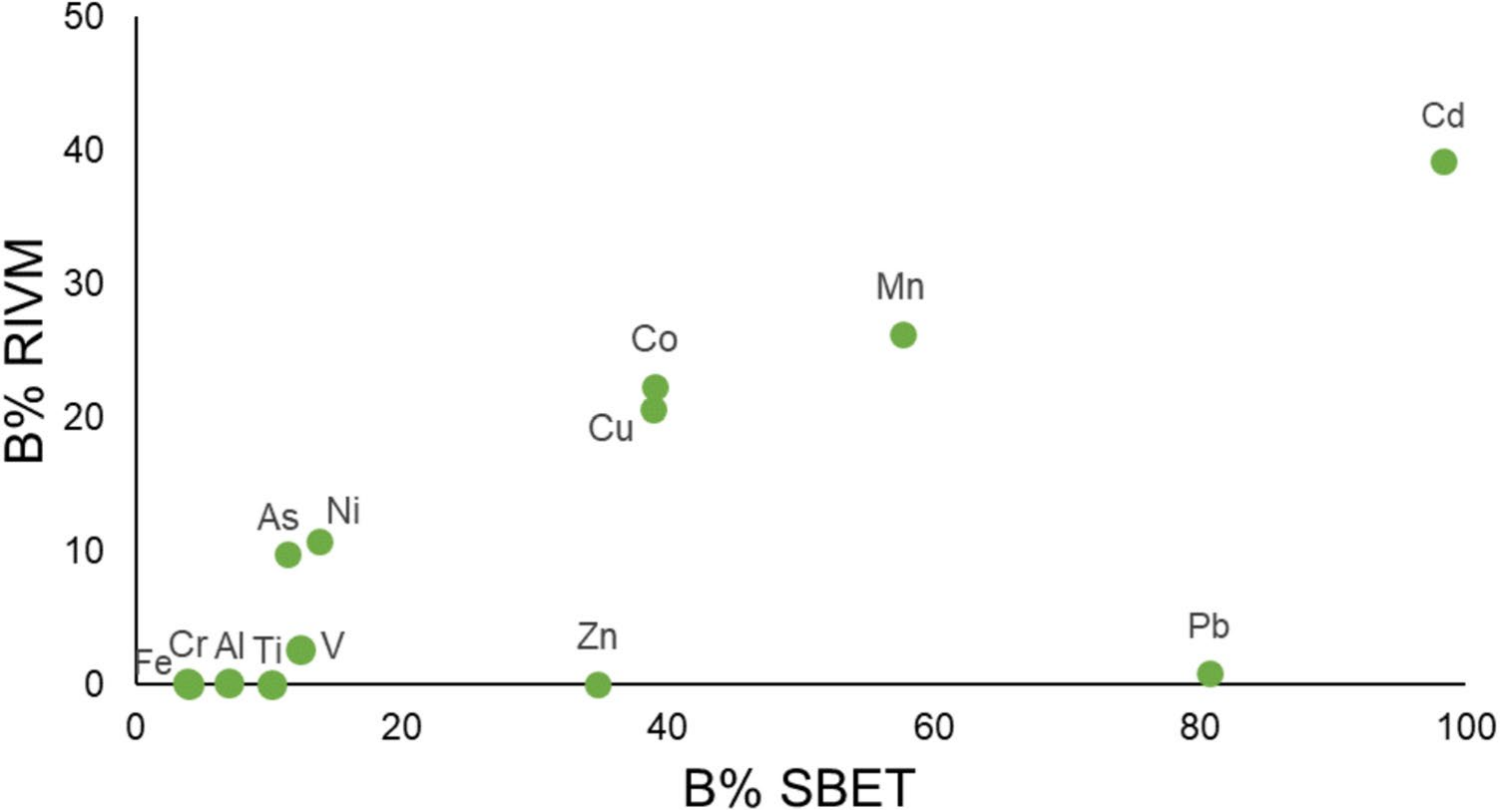
RIVM bioaccessibility (B% RIVM) vs SBET bioaccessibility (B% SBET)

Taking into account the average value of each metal/metalloid considering all the samples, in general, the order of bioaccessibility of metals and metalloids was quite similar in both methods (Fig. 5). For instance, SBET and RIVM extracted concentrations were further from the pseudototal in the case of Al, Cr, Fe, Ti and V, which means low bioaccessibility (3.9–12.4% in SBET and 0–3% in RIVM, on average). Ni and As showed a similar trend, but RIVM bioaccessibility was a bit higher, between 11 and 14%. In similar studies, As SBET bioaccessibility was around 4% (Ning et al 2021), and As solubility was found to be similar in the gastric and intestinal compartments (Ellickson et al 2001). On the other side, Cd concentration extracted by SBET and RIVM and the pseudototal value were very close to each other, leading to a high bioaccessibility in SBET (98%) and RIVM (39%). Co, Cu and Mn showed intermediate values for both methods: 38–58% for SBET and 20–30% for RIVM.

However, Pb and Zn showed different trends with each method. Pb was identified as the second highest bioaccessible element by SBET method (80.7%) and nearly the least bioaccessible by RIVM method (0.9%). Similar results were obtained for Pb bioaccessibility in other studies. For instance, Ellickson et al. (2001) stated an 86% decrease in Pb solubility as it moved from the gastric to the intestinal compartment. The difference between RIVM and SBET could be related to the metal resorption in the soil, pepsin complexion or chemical precipitation of metals due to the higher pH of intestinal juices (Roussel et al 2010). Pepsin catalyses the degradation of BSA, which generates agents that can reduce metal(loid) species. In addition, bile salts are known to increase interactions between metals leading to precipitation of the metal-bile complex and reduced bioaccessibility for some metal(loid) species (Alava et al. 2013). On the other hand, the percentage of Zn was 34.7% by SBET and almost 0% by RIVM. Zn low RIVM bioaccessibility values are due to the high amount of Zn of the blanks in RIVM method, so the bioaccessibility values are below the detection limit.

### The influence of soil properties in SBET/RIVM bioaccessibility (%)

Regarding the effect of soil properties of each area on bioaccessibility, phyllosilicates and the bioaccessibility (%) by SBET and RIVM were generally negatively correlated, and that correlation was statistically significant (at 95% confidence level) between phyllosilicates and Ni SBET B% (r = −0.516) (Table S4) and phyllosilicates and Pb RIVM B% (r = −0.528) (Table S5). This may indicate that in soils with a higher number of phyllosilicates metals are linked to phyllosilicates and that a smaller part is available for human absorption. According to a study which investigated the effect of soil mineralogy in oral bioaccessibility (calculated by SBET), carbonates disappeared from the XRD patterns during the bioaccessibility extraction testing, but, in contrast, quartz and phyllosilicates were found to be relatively stable during the extraction (Ettler et al. 2020).

**Fig. 6:**
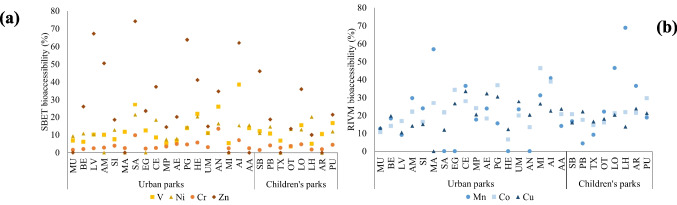
**a** SBET bioaccessibility of V and Ni in the studied urban parks. **b** RIVM bioaccessibility of Mn, Co and Cu in the studied urban parks

In general, a positive correlation has been observed between calcite content and SBET bioaccessibility for most of the metals and metalloids (Al, Ti, V, Cr, Mn, Fe, Co, Ni, Zn and As), but especially for V and Ni (r = 0.848 and r = 0.853, respectively, at 95% confidence level) (Table S4). In the studied soils, those with higher calcite content (AN, SA and HE) had higher SBET bioaccessibility of V and Ni (Table S2, Fig. 6a). A lower (but also positive) correlation was observed between calcite and Fe and Mn SBET bioaccessibilities (r = 0.536 and r = 0.528, at 95% confidence level) (Table S4). According to the review of Billmann et al (2023), metal(loid)s associated to carbonates are generally relatively bioaccessible due to the acid pH of the gastric phase. Cox et al. (2013) found that Ni is often linked to carbonates and the Ni bound to carbonates can be easily released in the gastric acid phase. This could explain the positive correlation between soil calcite content and Ni SBET bioaccessibility.

On the other side, there was a negative correlation between calcite and RIVM bioaccessibility for most of the metals and metalloids (V, Mn, Co, Ni, Cu, As and Pb), especially between calcite and the RIVM bioaccessibility of Co and Cu (r = −0.516 and r = −0.576) (Table S5). The soils with higher amount of calcite (AN, SA and HE) had lower RIVM bioaccessibility of Co and Cu (Table S2, Fig. 6b). Billmann et al (2023) reported that the total amount of carbonates is not always sufficient to understand bioaccessibility, due to the fact that some elements may have variable affinities depending on chemical speciation.

Cr and Zn SBET bioaccessibility was positively correlated with soil organic matter content (r = 0.544 for Cr and r = 0.530 for Zn) (Table S4). For instance, TX and AR contained less organic matter (8%) and Zn SBET bioaccessibility was zero; while LV, SA, PG and AI contained more organic matter (14–24%) and higher Zn SBET bioaccessibility (62–74%) (Table S2, Fig. 6a). Cr results go in accordance with the study conducted by Shi et al. (2023), who investigated the effects of soil organic carbon content on Cr in vitro bioaccessibility. They concluded that increasing soil organic carbon levels can reduce Cr bioaccessibility, because it promotes the reduction of Cr(VI) (actually toxic to humans) to Cr(III) (less toxic to humans) in the soils (Shi et al. 2023). Despite the significant positive correlation with Cr and Zn, there was no significant correlation between soil organic matter and other metal(loid)s SBET bioaccessibility. No significant correlations were observed either between RIVM bioaccessibility and soil organic matter (Table S5). Billmann et al (2023) reported that the literature showed that organic matter can decrease or increase the bioaccessibility of metals and metalloids in soils.

Regarding the correlations with soil pH, Cr SBET bioaccessibility was positively correlated (r = 0.680) (Table S4). This results are consistent with the study conducted by Shi et al. (2023), since they concluded that soil acidic conditions can reduce Cr bioaccessibility (Shi et al. 2023). In contrast, there was not statistically significant correlation between soil pH values and the SBET bioaccessibilty of the rest of the metal(loid)s. Izquierdo et al. (2015) who investigated the gastric bioaccessibility of metals in urban gardens of Madrid, reported that the lack of influence of soil pH on metal bioaccessibility could be explained by its narrow range of variation.

On the other hand, there was a significant negative correlation between soil pH and Mn RIVM bioaccessibility (r = −0.543) (Table S5). The soil samples with high values of pH, such as PB, TX, HE and AN, showed lower Mn RIVM bioaccessibility values (Table S2, Fig. 6b). Negative correlations between soil pH and metal bioaccessibility were found in the literature for gastric and intestinal phases. According to Billmann et al (2023), soil pH is a significant parameter that control the availability of metal(loid)s directly or indirectly by influencing other soil properties (such as the charge of Al and Fe oxides and the clay exchange capacity) and has a direct effect on absorption. A soil with a low pH can lead to the desorption of metals from organic compounds, clays or other sorbent compounds, while an alkaline pH can lead to a substantial adsorption, which can sometimes be irreversible (Billmann et al. 2023).

### Human health risk assessment

Hazard Quotient (HQ) and Carcinogenic Risk (CR) values were calculated by considering the bioaccessibility values obtained for each site and each element. Table S6 and Table S7 show the HQ and HI values in each site, for children and adults, respectively.

**Fig. 7:**
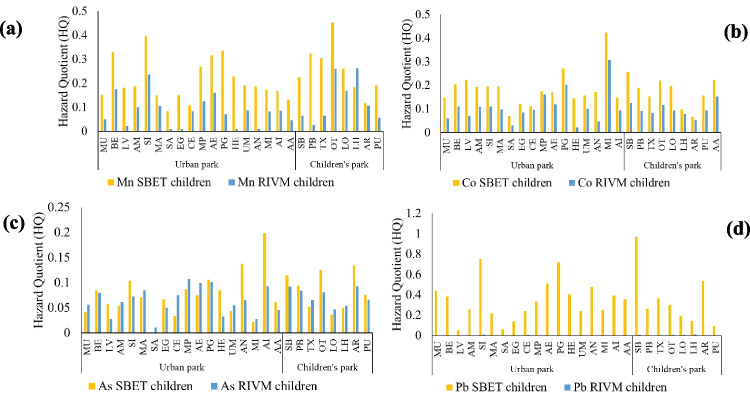
Hazard Quotients (HQ) for children for Mn (**a**), Co (**b**), As (**c**) and Pb (**d**) in the studied urban park soils, with the bioaccessibility value calculated by SBET and RIVM methodologies

HQ values for the metals and metalloids studied in this work (Mn, Cr, Fe, Co, Ni, Cu, Zn, As, Cd and Pb) were all below 1, thus, no adverse health effects are expected in the sampling sites. The risk for children is always higher than for adults. Lead showed the highest HQ value (for children, by SBET), with a maximum value in the sample PB (HQ = 0.97), which is a children’s park. The order for mean values of HQ was as follows: Pb > Mn > Co > As > Fe > Cu > Ni > Zn > Cd > Cr. In general, values calculated with SBET bioaccessibility values were always higher than those calculated by RIVM, except for Ni, since the RIVM value is higher in some samples. For Fe, Zn, Cd an Pb the difference between the HQ index calculated by the value of SBET and the value of RIVM is higher than in the case of Mn, Co, Ni, Cu and As. Figure 7 shows the HQ indexes for the elements with the highest values: Mn, Co, As and Pb.

Table 2 shows the HI and total CR values (by SBET and RIVM) for children. Regarding HI index (by SBET) for children, which considers the exposure and the cumulative effect of the 11 metal(loid)s mentioned, eight parks (BE, SI, AE, GP, AN, PB, OT and PU) were above 1 (Table 2). There is, therefore, a potential risk that non-carcinogenic effects may occur to children in these parks. Special attention should be paid to parks PB, OT and PU, due to the fact that they are children’s parks. HI index for adults (by SBET) was always below 1, so there is no significant risk of non-carcinogenic effects for adults (Table S7). HI calculated by RIVM was always below 1, for both children and adults (Table 2, Table S6 and Table S7).

**Table 2:** HI and Total CR values (by SBET and RIVM) for children in the studied 26 soils

		HI SBET	HI RIVM	Total CR SBET	Total CR RIVM
Urban park	MU	0.80	0.17	2.75E-06	2.16E-06
	BE	**1.06**	0.38	4.31E-06	3.09E-06
	LV	0.53	0.12	2.38E-06	1.10E-06
	AM	0.72	0.27	2.77E-06	2.36E-06
	SI	**1.50**	0.44	6.06E-06	2.82E-06
	MA	0.65	0.29	3.30E-06	3.29E-06
	SA	0.24	0.05	1.48E-06	4.05E-07
	EG	0.49	0.15	2.94E-06	1.92E-06
	CE	0.52	0.27	1.96E-06	2.92E-06
	MP	0.89	0.40	4.40E-06	4.14E-06
	AE	**1.11**	0.40	5.12E-06	3.83E-06
	GP	**1.47**	0.38	6.03E-06	3.90E-06
	HE	0.91	0.07	4.45E-06	1.24E-06
	UM	0.66	0.25	2.40E-06	2.13E-06
	AN	**1.06**	0.13	7.04E-06	2.50E-06
	MI	0.89	0.42	1.56E-06	1.08E-06
	AI	0.97	0.28	8.69E-06	3.59E-06
	AA	0.58	0.27	3.27E-06	1.75E-06
Children's park	SB	0.92	0.24	7.00E-06	3.57E-06
	PB	**1.64**	0.22	4.33E-06	3.24E-06
	TX	0.84	0.24	3.08E-06	2.52E-06
	OT	**1.14**	0.46	5.70E-06	3.12E-06
	LO	0.92	0.36	1.92E-06	1.81E-06
	LH	0.54	0.40	2.31E-06	2.12E-06
	AR	0.39	0.23	8.22E-06	3.60E-06
	PU	**1.07**	0.25	3.21E-06	2.54E-06

As expected, Carcinogenic Risk (CR) indexes calculated with the bioaccessibility values from SBET were generally higher than those calculated with RIVM. Table S8 and S9 show the individual (for Cr, As and Pb) and total CR values for children and adults. The Carcinogenic Risk individual levels of Cr, As and Pb for children in the sampling sites were lower than 1·10^–4^, thus, the carcinogenic risk of Cr, As and Pb of the studied urban park soils was within the tolerable range in the studied soils. The total carcinogenic risk (considering the sum of the above three elements) was also within the tolerable risk (below 1·10^–4^) for children (Table 2) and sometimes negligible (below 1·10^–6^) for adults (Table S9).

## Conclusions

More risks were always detected with SBET method rather than RIVM method, since higher bioaccessibility values were obtained. The difference could be due to the addition of a final intestinal step in RIVM which more closely simulates human digestion. That leads to more conservative result by means of SBET and, probably, more realistic ones with RIVM. According to HI index (by SBET), special attention should be paid to three children’s parks (PB, OT and PU), due to the fact that non-carcinogenic effects may occur to children in these parks. The total carcinogenic risk (considering Cr, As and Pb) was within the tolerable risk (below 1·10^–4^) for children and sometimes negligible (below 1·10^–6^) for adults.

Analysing the bioaccessibility percentages calculated for both methods and for each element, Cd showed the highest percentage of bioaccessibility in both methods and Fe, Al, Cr, Ti and V were the least bioaccessible elements. Pb and Zn showed different trends with each method.

Considering the effect of soil properties, there was a negative correlation between the content of phyllosillicates and the bioaccessibility by SBET and RIVM. On the other hand, there was a positive correlation between calcite and SBET bioaccessibility for most of the metals, except for Cu, Cd and Pb. In contrast, there was a negative correlation between calcite and RIVM bioaccessibility for most of the metals. Soil pH did not influence that much on SBET bioaccessibility but it did on RIVM bioaccessibility, especially in Mn bioaccessibility by RIVM.

Taking into account the experimental complexity and the small difference between methods for most of the metals, SBET method may be used for performing a simple and conservative first approach of the bioaccessibility of metals and metalloids due to the ingest of soils. Nonetheless, considering the different results obtained for Pb and Zn by both methods, and the possibility of overestimating the risk by SBET, it would be advisable to follow both methods to determine the bioaccessibility of Pb and Zn.

## Supplementary information

Below is the link to the electronic supplementary material.

ESM 1 (DOCX 15.1 KB)

ESM 2 (DOCX 20.0 KB)

ESM 3 (DOCX 18.4 KB)

ESM 4 (DOCX 17.3 KB)

ESM 5 (DOCX 15.3 KB)

ESM 6 (DOCX 21.2 KB)

ESM 7 (DOCX 20.3 KB )

ESM 8 (DOCX 17.3 KB)

ESM 9 (DOCX 17.8 KB)

## Data Availability

Data will be made available on reasonable request.
