# Supplementary material for: Simplified vs extended in vitro methods for the evaluation of bioaccessibility of metals and metalloids present in urban recreational soils
Source: Environ Sci Pollut Res Int. 2025 Feb 9;32(9):5358–70. doi: 10.1007/s11356-025-36017-y (PMC11868185; doi:10.1007/s11356-025-36017-y)
Supplement: Supplementary file 1 — (DOCX 15.1 KB) [file 11356_2025_36017_MOESM1_ESM.docx]

**Supplementary Table 1.** Composition of the synthetic juices of the RIVM *in vitro* digestion (Oomen et al., 2003).

|  | Saliva | Gastric juice | Duodenal juice | Bile |
| --- | --- | --- | --- | --- |
| Inorganic solution | 10 mL KCl 89.6 g/L  10 mL KSCN 20 g/L  10 mL NaH_2_PO_4_ 88.8 g/L  10 mL Na_2_PO_4_ 57 g/L  1.7 mL NaCl 175.3 g/L  1.8 mL NaOH 40 g/L | 15.7 mL NaCl 175.3 g/L  3.0 mL NaH_2_PO_4_ 88.8 g/L  9.2 mL KCl 89.6 g/L  18 mL CaCl_2_ · 2H_2_O 22.2 g/L  10 mL NH_4_Cl 30.6 g/L  8.3 mL HCl %37 g/g | 40 mL NaCl 175.3 g/L  40 mL NaHCO_3_ 84.7 g/L  10 mL KH_2_PO_4_ 8 g/L  6.3 mL KCl 89.6 g/L  10 mL MgCl_2_ 5 g/L  180 µL HCl %37 g/g | 30 mL NaCl 175.3 g/L  68.3 mL NaHCO_3_ 84.7 g/L  4.2 mL KCl 89.6 g/L  200 µL HCl %37 g/g |
| Organic solution | 8 mL urea 25 g/L | 10 mL glucose 65 g/L  10 mL glucuronic acid 2 g/L  3.4 mL urea 25 g/L  10 mL glucosamine hydrochloride 33 g/L | 4 mL urea 25 g/L | 10 mL urea 25 g/L |
| Additional substances | 145 mg α-amilase  15 mg uric acid  50 mg mucin | 1 g BSA^a^  1 g pepsin  3 g mucin | 9 mL CaCl_2_ · 2H_2_O 22.2 g/L  1 g BSA^a^  3 g pancreatin  0.5 g lipase | 10 mL CaCl_2_ · 2H_2_O 22.2 g/L  1.8 g BSA^a^  6 g bile |
| pH | 6.5 ± 0.2 | 1.07 ± 0.07 | 7.8 ± 0.2 | 8.0 ± 0.2 |

^a^Bovine Serum Albumine
