# Supplementary material for: Simplified vs extended in vitro methods for the evaluation of bioaccessibility of metals and metalloids present in urban recreational soils
Source: Environ Sci Pollut Res Int. 2025 Feb 9;32(9):5358–70. doi: 10.1007/s11356-025-36017-y (PMC11868185; doi:10.1007/s11356-025-36017-y)
Supplement: Supplementary file 2 — (DOCX 20.0 KB) [file 11356_2025_36017_MOESM2_ESM.docx]

**Supplementary Table 2**. Physicochemical properties and mineralogy of the studied 26 urban park soils of San Sebastian.

|  | Sample | pH | OM (%) | Soil mineralogy (%) | | | | Clay minerals (%) | | | | |
| --- | --- | --- | --- | --- | --- | --- | --- | --- | --- | --- | --- | --- |
|  |  |  |  | **Quartz** | **Calcite** | **Phyllosil.** | **Felds.** | **Illite** | **Kaolinite** | **Chlorites** | **Vermiculite** | **Smectite** |
| Urban park | **MU** | 7.62 | 15.67 | 41 |  | 59 |  | 87 | 5 | 8 |  |  |
|  | **BE** | 6.84 | 11.56 | 48 |  | 52 |  | 82 | 18 |  |  |  |
|  | **LV** | 7.68 | 16.69 | 38 | 4 | 58 |  | 93 | 3 | 4 |  |  |
|  | **AM** | 7.17 | 17.45 | 53 |  | 47 |  | 84 |  | 16 |  |  |
|  | **OT** | 6.61 | 10.53 | 59 |  | 41 |  | 84 | 5 | 11 |  |  |
|  | **SI** | 6.79 | 13.73 | 43 |  | 57 |  | 85 |  | 15 |  |  |
|  | **LO** | 7.33 | 13.02 | 58 | 3 | 39 |  | 84 |  | 16 |  |  |
|  | **MA** | 6.78 | 24.54 | 47 |  | 53 |  | 83 | 3 | 9 | 5 |  |
|  | **LH** | 6.14 | 11.85 | 68 |  | 32 |  | 84 | 7 | 9 |  |  |
|  | **SA** | 7.26 | 13.63 | 46 | 30 | 24 |  | 82 | 8 |  |  | 10 |
|  | **AE** | 7.24 | 12.58 | 56 | 3 | 41 |  | 80 |  | 20 |  |  |
|  | **PG** | 8.04 | 15.31 | 62 | 4 | 34 |  | 85 | 8 | 7 |  |  |
|  | **HE** | 8.18 | 21.8 | 20 | 39 | 41 |  | 69 | 16 | 15 |  |  |
|  | **UM** | 8.07 | 10.07 | 48 | 3 | 47 | 2 | 85 | 6 | 9 |  |  |
|  | **AN** | 7.87 | 12.59 | 43 | 25 | 32 |  | 75 | 7 | 6 | 12 |  |
|  | **MI** | 5.73 | 11.88 | 63 |  | 36 | 1 | 69 | 6 | 7 | 18 |  |
|  | **AI** | 7.03 | 24.39 | 66 |  | 34 |  | 86 | 5 | 9 |  |  |
|  | **AA** | 6.33 | 20.07 | 37 |  | 63 |  | 97 | 3 |  |  |  |
| Children's park | **SB** | 7.38 | 15.38 | 54 |  | 46 |  | 77 | 23 |  |  |  |
|  | **PB** | 7.87 | 11.72 | 56 | 3 | 41 |  | 84 |  | 16 |  |  |
|  | **TX** | 7.95 | 8.12 | 56 | 5 | 39 |  | 78 |  | 22 |  |  |
|  | **EG** | 7.42 | 11.91 | 74 | 2 | 24 |  | 96 |  | 4 |  |  |
|  | **CE** | 6.55 | 14.54 | 48 |  | 52 |  | 85 | 6 | 9 |  |  |
|  | **MP** | 7.24 | 17.3 | 43 |  | 57 |  | 81 |  | 19 |  |  |
|  | **AR** | 6.37 | 8.17 | 79 |  | 19 | 2 | 75 |  | 25 |  |  |
|  | **PU** | 7.06 | 15.91 | 67 |  | 33 |  | 87 | 5 | 8 |  |  |
|  | **MIN.** | **5.73** | **8.12** | **20** | **2** | **19** | **1** | **69** | **3** | **4** | **5** | **10** |
|  | **MAX.** | **8.18** | **24.54** | **79** | **39** | **63** | **2** | **97** | **23** | **25** | **18** | **10** |
|  | **MEAN** | **7.17** | **14.63** | **53** | **11** | **42** | **2** | **83** | **8** | **12** | **12** | **10** |
