# Supplementary material for: Simplified vs extended in vitro methods for the evaluation of bioaccessibility of metals and metalloids present in urban recreational soils
Source: Environ Sci Pollut Res Int. 2025 Feb 9;32(9):5358–70. doi: 10.1007/s11356-025-36017-y (PMC11868185; doi:10.1007/s11356-025-36017-y)
Supplement: Supplementary file 3 — (DOCX 18.4 KB) [file 11356_2025_36017_MOESM3_ESM.docx]

**Supplementary Table 3**. Correlation coefficient (r) or Pearson correlation factor between the soil properties and pseudototal metal/metalloid concentrations.

|  | pH | OM (%) | Q (%) | C (%) | P (%) | Illi. (%) | Kao. (%) | Chl, (%) | Al | Ti | V | Cr | Mn | Fe | Co | Ni | Cu | Zn | As | Cd | Pb |
| --- | --- | --- | --- | --- | --- | --- | --- | --- | --- | --- | --- | --- | --- | --- | --- | --- | --- | --- | --- | --- | --- |
| pH | 1 |  |  |  |  |  |  |  |  |  |  |  |  |  |  |  |  |  |  |  |  |
| OM (%) | 0.023 | 1 |  |  |  |  |  |  |  |  |  |  |  |  |  |  |  |  |  |  |  |
| Q (%) | -0.354 | -0.420 | 1 |  |  |  |  |  |  |  |  |  |  |  |  |  |  |  |  |  |  |
| C (%) | 0.182 | **0.589** | *-0.741* | 1 |  |  |  |  |  |  |  |  |  |  |  |  |  |  |  |  |  |
| P (%) | 0.002 | 0.371 | *-0.667* | -0.294 | 1 |  |  |  |  |  |  |  |  |  |  |  |  |  |  |  |  |
| Illi. (%) | -0.001 | 0.166 | 0.069 | *-0.689* | 0.302 | 1 |  |  |  |  |  |  |  |  |  |  |  |  |  |  |  |
| Cao. (%) | 0.231 | -0.124 | -0.160 | **0.762** | -0.078 | -0.518 | 1 |  |  |  |  |  |  |  |  |  |  |  |  |  |  |
| Clo. (%) | -0.007 | -0.263 | 0.055 | -0.004 | -0.069 | -0.431 | 0.688 | 1 |  |  |  |  |  |  |  |  |  |  |  |  |  |
| Al | -0.007 | -0.175 | -0.197 | -0.278 | 0.423 | -0.195 | 0.160 | 0.060 | 1 |  |  |  |  |  |  |  |  |  |  |  |  |
| Ti | -0.046 | -0.382 | 0.081 | 0.174 | -0.089 | -0.393 | 0.310 | 0.396 | **0.609** | 1 |  |  |  |  |  |  |  |  |  |  |  |
| V | 0.202 | 0.109 | -0.465 | 0.447 | 0.319 | -0.465 | **0.506** | -0.088 | **0.767** | 0.443 | 1 |  |  |  |  |  |  |  |  |  |  |
| Cr | 0.187 | -0.216 | -0.332 | 0.032 | 0.311 | -0.300 | 0.387 | 0.250 | **0.770** | **0.758** | **0.706** | 1 |  |  |  |  |  |  |  |  |  |
| Mn | -0.048 | -0.362 | -0.051 | -0.490 | 0.304 | -0.045 | 0.249 | 0.408 | **0.627** | **0.747** | 0.325 | **0.645** | 1 |  |  |  |  |  |  |  |  |
| Fe | -0.006 | -0.236 | -0.318 | -0.254 | **0.512** | -0.020 | 0.169 | 0.229 | **0.671** | **0.573** | 0.491 | **0.722** | **0.852** | 1 |  |  |  |  |  |  |  |
| Co | -0.135 | -0.167 | -0.242 | *-0.587* | **0.613** | -0.021 | 0.218 | 0.241 | **0.675** | 0.449 | 0.453 | **0.637** | **0.747** | **0.839** | 1 |  |  |  |  |  |  |
| Ni | 0.143 | -0.088 | *-0.521* | -0.251 | **0.665** | 0.244 | 0.127 | 0.170 | 0.486 | 0.345 | 0.355 | **0.636** | **0.658** | **0.860** | **0.761** | 1 |  |  |  |  |  |
| Cu | 0.209 | -0.236 | -0.099 | -0.261 | 0.168 | -0.057 | 0.337 | 0.374 | 0.448 | **0.529** | 0.326 | **0.792** | **0.572** | **0.574** | **0.511** | **0.590** | 1 |  |  |  |  |
| Zn | 0.272 | -0.141 | -0.232 | 0.092 | 0.195 | -0.061 | 0.334 | 0.348 | 0.260 | 0.442 | 0.284 | 0.477 | **0.548** | 0.451 | 0.366 | 0.404 | 0.445 | 1 |  |  |  |
| As | -0.004 | -0.149 | -0.161 | -0.334 | 0.434 | -0.054 | 0.178 | 0.393 | **0.665** | **0.595** | 0.448 | **0.626** | **0.914** | **0.859** | **0.748** | **0.665** | **0.562** | **0.515** | 1 |  |  |
| Cd | **0.504** | 0.102 | *-0.507* | **0.645** | 0.027 | -0.244 | 0.276 | 0.076 | 0.047 | 0.232 | 0.383 | 0.359 | 0.229 | 0.377 | 0.066 | 0.402 | 0.265 | **0.511** | 0.299 | 1 |  |
| Pb | 0.339 | -0.076 | -0.091 | -0.117 | 0.163 | -0.126 | 0.341 | 0.173 | 0.371 | 0.401 | 0.368 | 0.398 | **0.548** | 0.448 | 0.374 | 0.337 | 0.377 | **0.644** | **0.608** | **0.577** | 1 |

*Q=Quartz, C=Calcite, P=Phyllosilicates, Illi.=Illite, Kao.=Kaolinite, Chl.=Chlorites.

**Significant positive correlation** > r=0.496 (N=26, 0.01 two-tailed, at 95% confidence level)

*Significant negative correlation* < r=-0.496 (N=26, 0.01 two-tailed, at 95% confidence level)
