# Supplementary material for: Simplified vs extended in vitro methods for the evaluation of bioaccessibility of metals and metalloids present in urban recreational soils
Source: Environ Sci Pollut Res Int. 2025 Feb 9;32(9):5358–70. doi: 10.1007/s11356-025-36017-y (PMC11868185; doi:10.1007/s11356-025-36017-y)
Supplement: Supplementary file 4 — (DOCX 17.3 KB) [file 11356_2025_36017_MOESM4_ESM.docx]

|  | Al% SBET | Ti% SBET | V% SBET | Cr% SBET | Mn% SBET | Fe% SBET | Co% SBET | Ni% SBET | Cu% SBET | Zn% SBET | As% SBET | Cd% SBET | Pb% SBET | Q (%) | C (%) | P (%) | pH | OM (%) |
| --- | --- | --- | --- | --- | --- | --- | --- | --- | --- | --- | --- | --- | --- | --- | --- | --- | --- | --- |
| Al% SBET | 1 |  |  |  |  |  |  |  |  |  |  |  |  |  |  |  |  |  |
| Ti% SBET | **0.759** | 1 |  |  |  |  |  |  |  |  |  |  |  |  |  |  |  |  |
| V% SBET | **0.789** | **0.653** | 1 |  |  |  |  |  |  |  |  |  |  |  |  |  |  |  |
| Mn% SBET | **0.551** | 0.481 | **0.705** | 1 | 1 |  |  |  |  |  |  |  |  |  |  |  |  |  |
| Fe% SBET | **0.780** | **0.744** | **0.785** | **0.544** | 0.414 | 1 |  |  |  |  |  |  |  |  |  |  |  |  |
| Co% SBET | **0.534** | 0.395 | **0.570** | **0.580** | **0.883** | 0.479 | 1 |  |  |  |  |  |  |  |  |  |  |  |
| Ni% SBET | **0.516** | 0.266 | 0.467 | 0.334 | 0.444 | 0.297 | 0.292 | 1 |  |  |  |  |  |  |  |  |  |  |
| Cu% SBET | 0.471 | **0.536** | 0.469 | 0.395 | 0.373 | **0.536** | 0.309 | 0.166 | 1 |  |  |  |  |  |  |  |  |  |
| Zn% SBET | **0.529** | 0.218 | **0.595** | 0.392 | **0.701** | 0.251 | **0.720** | 0.309 | -0.041 | 1 |  |  |  |  |  |  |  |  |
| As% SBET | **0.696** | **0.574** | **0.913** | 0.314 | **0.625** | **0.807** | **0.565** | 0.144 | **0.650** | 0.484 | 1 |  |  |  |  |  |  |  |
| Cd% SBET | -0.127 | -0.119 | -0.106 | **0.582** | -0.105 | -0.156 | -0.148 | -0.207 | 0.020 | 0.085 | 0.013 | 1 |  |  |  |  |  |  |
| Pb% SBET | 0.075 | 0.148 | 0.146 | -0.139 | 0.202 | 0.263 | 0.276 | -0.179 | **0.516** | -0.188 | 0.312 | -0.003 | 1 |  |  |  |  |  |
| Q (%) | 0.080 | -0.075 | -0.068 | 0.175 | 0.004 | 0.144 | 0.140 | -0.069 | 0.397 | -0.182 | 0.224 | 0.477 | 0.291 | 1 |  |  |  |  |
| C (%) | 0.470 | 0.427 | **0.848** | 0.330 | **0.528** | **0.536** | 0.238 | **0.853** | -0.229 | 0.351 | 0.219 | -0.385 | -0.182 | *-0.741* | 1 |  |  |  |
| P (%) | -0.355 | -0.296 | -0.380 | 0.093 | -0.417 | -0.283 | -0.314 | *-0.516* | -0.384 | -0.157 | -0.375 | -0.414 | -0.247 | *-0.667* | -0.294 | 1 |  |  |
| pH | -0.109 | 0.147 | 0.276 | **0.680** | 0.302 | -0.138 | 0.027 | -0.148 | 0.045 | 0.299 | 0.238 | -0.215 | -0.061 | -0.354 | 0.182 | 0.002 | 1 |  |
| OM (%) | 0.237 | 0.115 | 0.476 | **0.544** | 0.413 | 0.366 | 0.424 | 0.151 | 0.108 | **0.530** | 0.397 | -0.244 | 0.071 | -0.420 | **0.589** | 0.371 | 0.023 | 1 |

**Supplementary Table 4**. Correlation coefficient (r) or Pearson correlation factor between the soil properties and SBET bioaccessibility % of the studied metals/metalloids.

*Q=Quartz, C=Calcite, P=Phyllosilicates.

**Significant positive correlation** > r=0.496 (N=26, 0.01 two-tailed, at 95% confidence level)

*Significant negative correlation* < r=-0.496 (N=26, 0.01 two-tailed, at 95% confidence level)
