# Supplementary material for: Simplified vs extended in vitro methods for the evaluation of bioaccessibility of metals and metalloids present in urban recreational soils
Source: Environ Sci Pollut Res Int. 2025 Feb 9;32(9):5358–70. doi: 10.1007/s11356-025-36017-y (PMC11868185; doi:10.1007/s11356-025-36017-y)
Supplement: Supplementary file 5 — (DOCX 15.3 KB) [file 11356_2025_36017_MOESM5_ESM.docx]

**Supplementary Table 5**. Correlation coefficient (r) or Pearson correlation factor between the soil properties and RIVM bioaccessibility % of the studied metals/metalloids.

|  | Al% RIVM | V% RIVM | Mn% RIVM | Co% RIVM | Ni% RIVM | Cu% RIVM | As% RIVM | Pb% RIVM | Q (%) | C (%) | P (%) | pH | OM (%) |
| --- | --- | --- | --- | --- | --- | --- | --- | --- | --- | --- | --- | --- | --- |
| Al% RIVM | 1 |  |  |  |  |  |  |  |  |  |  |  |  |
| V% RIVM | 0.463 | 1 |  |  |  |  |  |  |  |  |  |  |  |
| Mn% RIVM | 0.387 | 0.201 | 1 |  |  |  |  |  |  |  |  |  |  |
| Co% RIVM | 0.005 | 0.405 | 0.319 | 1 |  |  |  |  |  |  |  |  |  |
| Ni% RIVM | -0.108 | -0.029 | 0.012 | 0.237 | 1 |  |  |  |  |  |  |  |  |
| Cu% RIVM | 0.087 | 0.327 | 0.056 | **0.536** | 0.436 | 1 |  |  |  |  |  |  |  |
| As% RIVM | 0.263 | **0.722** | 0.224 | 0.452 | -0.088 | 0.326 | 1 |  |  |  |  |  |  |
| Pb% RIVM | **0.980** | 0.402 | 0.370 | -0.050 | -0.063 | 0.094 | 0.260 | 1 |  |  |  |  |  |
| Q (%) | 0.449 | 0.405 | 0.393 | **0.590** | -0.013 | 0.389 | 0.394 | **0.530** | 1 |  |  |  |  |
| C (%) | 0.289 | -0.323 | -0.478 | *-0.516* | -0.111 | *-0.576* | -0.075 | -0.420 | *-0.741* | 1 |  |  |  |
| P (%) | -0.414 | -0.311 | -0.331 | -0.328 | -0.016 | -0.157 | -0.442 | *-0.529* | *-0.667* | -0.294 | 1 |  |  |
| pH | -0.430 | -0.130 | *-0.543* | -0.403 | -0.033 | -0.145 | 0.057 | -0.357 | -0.354 | 0.182 | 0.002 | 1 |  |
| OM (%) | -0.245 | 0.269 | 0.200 | 0.151 | -0.327 | -0.182 | 0.208 | -0.340 | -0.420 | **0.589** | 0.371 | 0.023 | 1 |

*Q=Quartz, C=Calcite, P=Phyllosilicates.

**Significant positive correlation** > r=0.496 (N=26, 0.01 two-tailed, at 95% confidence level)

*Significant negative correlation* < r=-0.496 (N=26, 0.01 two-tailed, at 95% confidence level)
