# Supplementary material for: Simplified vs extended in vitro methods for the evaluation of bioaccessibility of metals and metalloids present in urban recreational soils
Source: Environ Sci Pollut Res Int. 2025 Feb 9;32(9):5358–70. doi: 10.1007/s11356-025-36017-y (PMC11868185; doi:10.1007/s11356-025-36017-y)
Supplement: Supplementary file 6 — (DOCX 21.2 KB) [file 11356_2025_36017_MOESM6_ESM.docx]

**Supplementary Table 6**. HQ index (for children) of 10 metal(loid)s analysed in the studied 26 urban soils and HI index of each soil (calculated as the sum of the 10 metal(loid) HQ values). (HQ or HI values < 0.005 expressed by "-")

|  |  | SBET HQ children | | | | | | | | | | | RIVM HQ children | | | | | | | | | | |
| --- | --- | --- | --- | --- | --- | --- | --- | --- | --- | --- | --- | --- | --- | --- | --- | --- | --- | --- | --- | --- | --- | --- | --- |
|  |  | **Mn** | **Fe** | **Co** | **Ni** | **Cu** | **Zn** | **As** | **Cd** | **Pb** | **Cr** | **HI** | **Mn** | **Fe** | **Co** | **Ni** | **Cu** | **Zn** | **As** | **Cd** | **Pb** | **Cr** | **HI** |
| Urban park | **MU** | 0.15 | 0.01 | 0.15 | - | - | - | 0.04 | 0.01 | 0.44 | - | **0.80** | 0.05 | - | 0.06 | - | - | - | 0.06 | - | - | - | **0.17** |
|  | **BE** | 0.33 | 0.04 | 0.21 | - | 0.01 | - | 0.08 | 0.01 | 0.38 | - | **1.06** | 0.17 | - | 0.11 | - | - | - | 0.08 | - | - | - | **0.38** |
|  | **LV** | 0.18 | 0.01 | 0.22 | - | - | - | 0.06 | - | 0.05 | - | **0.53** | 0.02 | - | 0.07 | - | - | - | 0.03 | - | - | - | **0.12** |
|  | **AM** | 0.19 | 0.01 | 0.19 | - | - | 0.01 | 0.05 | - | 0.26 | - | **0.72** | 0.10 | - | 0.11 | - | - | - | 0.06 | - | - | - | **0.27** |
|  | **SI** | 0.40 | 0.03 | 0.20 | - | 0.01 | - | 0.10 | 0.01 | 0.75 | - | **1.50** | 0.24 | - | 0.11 | - | - | - | 0.07 | - | 0.01 | - | **0.44** |
|  | **MA** | 0.15 | 0.02 | 0.20 | - | - | - | 0.07 | - | 0.22 | - | **0.65** | 0.11 | - | 0.10 | - | - | - | 0.09 | - | - | - | **0.29** |
|  | **SA** | 0.08 | 0.01 | 0.07 | - | - | 0.01 |  | 0.01 | 0.06 | - | **0.24** | 0.01 | - | 0.03 | - | - | - | 0.01 | - | - | - | **0.05** |
|  | **EG** | 0.15 | 0.01 | 0.12 | - | - | - | 0.07 | - | 0.14 | - | **0.49** | 0.01 | - | 0.08 | - | - | - | 0.05 | - | - | - | **0.15** |
|  | **CE** | 0.11 | 0.01 | 0.11 | - | - | - | 0.03 | - | 0.24 | - | **0.52** | 0.08 | - | 0.10 | - | - | - | 0.08 | - | - | - | **0.27** |
|  | **MP** | 0.27 | 0.01 | 0.17 | - | 0.01 | - | 0.09 | 0.01 | 0.33 | - | **0.89** | 0.12 | - | 0.16 | - | - | - | 0.11 | - | - | - | **0.40** |
|  | **AE** | 0.32 | 0.01 | 0.17 | - | 0.02 | - | 0.08 | 0.01 | 0.51 | - | **1.11** | 0.16 | - | 0.12 | - | 0.02 | - | 0.10 | - | - | - | **0.40** |
|  | **GP** | 0.34 | 0.01 | 0.27 | - | 0.01 | 0.01 | 0.11 | 0.01 | 0.72 | - | **1.47** | 0.07 | - | 0.20 | - | 0.01 | - | 0.10 | - | - | - | **0.38** |
|  | **HE** | 0.23 | 0.02 | 0.15 | - | - | - | 0.08 | 0.02 | 0.41 | - | **0.91** | 0.01 | - | 0.02 | - | - | - | 0.03 | - | - | - | **0.07** |
|  | **UM** | 0.19 | 0.01 | 0.16 | - | 0.01 | - | 0.04 | 0.01 | 0.24 | - | **0.66** | 0.09 | - | 0.10 | 0.01 | 0.01 | - | 0.06 | - | - | - | **0.25** |
|  | **AN** | 0.19 | 0.05 | 0.17 | - | 0.01 | - | 0.14 | 0.02 | 0.48 | - | **1.06** | 0.01 | - | 0.05 | - | - | - | 0.06 | - | - | - | **0.13** |
|  | **MI** | 0.17 | 0.02 | 0.42 | - | - | - | 0.02 | - | 0.25 | - | **0.89** | 0.08 | - | 0.31 | - | - | - | 0.03 | - | - | - | **0.42** |
|  | **AI** | 0.17 | 0.04 | 0.15 | - | - | - | 0.20 | 0.01 | 0.39 | - | **0.97** | 0.09 | - | 0.09 | - | - | - | 0.09 | - | - | - | **0.28** |
|  | **AA** | 0.13 | 0.04 | 0.22 | - | 0.01 | - | 0.08 | 0.01 | 0.09 | - | **0.58** | 0.05 | - | 0.15 | - | - | - | 0.07 | - | - | - | **0.27** |
| Children's park | **SB** | 0.23 | 0.01 | 0.26 | - | 0.01 | - | 0.06 | - | 0.35 | - | **0.92** | 0.06 | - | 0.12 | - | - | - | 0.05 | - | - | - | **0.24** |
|  | **PB** | 0.32 | 0.02 | 0.19 | - | 0.01 | - | 0.12 | 0.01 | 0.97 | - | **1.64** | 0.03 | - | 0.09 | - | - | - | 0.09 | - | 0.01 | - | **0.22** |
|  | **TX** | 0.30 | 0.01 | 0.15 | - | 0.01 | - | 0.09 | - | 0.26 | - | **0.84** | 0.06 | - | 0.08 | - | - | - | 0.08 | - | - | - | **0.24** |
|  | **OT** | 0.45 | 0.03 | 0.22 | - | 0.01 | - | 0.05 | 0.01 | 0.36 | - | **1.14** | 0.26 | - | 0.12 | - | - | - | 0.07 | - | - | - | **0.46** |
|  | **LO** | 0.26 | 0.02 | 0.20 | - | 0.01 | - | 0.13 | 0.01 | 0.30 | - | **0.92** | 0.17 | - | 0.09 | - | - | - | 0.08 | - | 0.01 | - | **0.36** |
|  | **LH** | 0.18 | 0.02 | 0.10 | - | 0.01 | - | 0.04 | 0.01 | 0.19 | - | **0.54** | 0.26 | - | 0.08 | - | - | - | 0.05 | - | - | - | **0.40** |
|  | **AR** | 0.12 | 0.01 | 0.07 | - | - | - | 0.05 | - | 0.14 | - | **0.39** | 0.11 | - | 0.05 | - | - | - | 0.05 | - | 0.01 | - | **0.23** |
|  | **PU** | 0.19 | 0.03 | 0.16 | - | - | - | 0.13 | 0.01 | 0.54 | - | **1.07** | 0.06 | - | 0.09 | - | - | - | 0.09 | - | - | - | **0.25** |
