# Supplementary material for: Simplified vs extended in vitro methods for the evaluation of bioaccessibility of metals and metalloids present in urban recreational soils
Source: Environ Sci Pollut Res Int. 2025 Feb 9;32(9):5358–70. doi: 10.1007/s11356-025-36017-y (PMC11868185; doi:10.1007/s11356-025-36017-y)
Supplement: Supplementary file 7 — (DOCX 20.3 KB ) [file 11356_2025_36017_MOESM7_ESM.docx]

**Supplementary Table 7**. HQ index (for adults) of 10 metal(loid)s analysed in the studied 26 urban soils and HI index of each soil (calculated as the sum of the 10 metal(loid) HQ values). (HQ or HI values < 0.005 expressed by "-")

|  |  | SBET HQ adult | | | | | | | | | | | RIVM HQ adult | | | | | | | | | | |  |
| --- | --- | --- | --- | --- | --- | --- | --- | --- | --- | --- | --- | --- | --- | --- | --- | --- | --- | --- | --- | --- | --- | --- | --- | --- |
|  |  | **Mn** | **Fe** | **Co** | **Ni** | **Cu** | **Zn** | **As** | **Cd** | **Pb** | **Cr** | **HI** | **Mn** | **Fe** | **Co** | **Ni** | **Cu** | **Zn** | **As** | **Cd** | **Pb** | **Cr** | **HI** | |
| Urban park | **MU** | 0.01 | - | 0.01 | - | - | - | - | - | 0.04 | - | **0.07** | 0.00 | - | 0.01 | - | - | - | 0.00 | - | - | - | **0.01** | |
|  | **BE** | 0.03 | - | 0.02 | - | - | - | 0.01 | - | 0.03 | - | **0.09** | 0.01 | - | 0.01 | - | - | - | 0.01 | - | - | - | **0.03** | |
|  | **LV** | 0.02 | - | 0.02 | - | - | - | - | - | - | - | **0.05** | 0.00 | - | 0.01 | - | - | - | 0.00 | - | - | - | **0.01** | |
|  | **AM** | 0.02 | - | 0.02 | - | - | - | - | - | 0.02 | - | **0.06** | 0.01 | - | 0.01 | - | - | - | 0.01 | - | - | - | **0.02** | |
|  | **SI** | 0.03 | - | 0.02 | - | - | - | 0.01 | - | 0.06 | - | **0.13** | 0.02 | - | 0.01 | - | - | - | 0.01 | - | - | - | **0.04** | |
|  | **MA** | 0.01 | - | 0.02 | - | - | - | 0.01 | - | 0.02 | - | **0.06** | 0.01 | - | 0.01 | - | - | - | 0.01 | - | - | - | **0.03** | |
|  | **SA** | 0.01 | - | 0.01 | - | - | - | - | - | 0.01 | - | **0.02** | 0.00 | - | 0.00 | - | - | - | 0.00 | - | - | - | **0.00** | |
|  | **EG** | 0.01 | - | 0.01 | - | - | - | 0.01 | - | 0.01 | - | **0.04** | 0.00 | - | 0.01 | - | - | - | 0.00 | - | - | - | **0.01** | |
|  | **CE** | 0.01 | - | 0.01 | - | - | - | - | - | 0.02 | - | **0.04** | 0.01 | - | 0.01 | - | - | - | 0.01 | - | - | - | **0.02** | |
|  | **MP** | 0.02 | - | 0.01 | - | - | - | 0.01 | - | 0.03 | - | **0.08** | 0.01 | - | 0.01 | - | - | - | 0.01 | - | - | - | **0.03** | |
|  | **AE** | 0.03 | - | 0.01 | - | - | - | 0.01 | - | 0.04 | - | **0.10** | 0.01 | - | 0.01 | - | - | - | 0.01 | - | - | - | **0.03** | |
|  | **GP** | 0.03 | - | 0.02 | - | - | - | 0.01 | - | 0.06 | - | **0.13** | 0.01 | - | 0.02 | - | - | - | 0.01 | - | - | - | **0.03** | |
|  | **HE** | 0.02 | - | 0.01 | - | - | - | 0.01 | - | 0.03 | - | **0.08** | 0.00 | - | 0.00 | - | - | - | 0.00 | - | - | - | **0.01** | |
|  | **UM** | 0.02 | - | 0.01 | - | - | - | - | - | 0.02 | - | **0.06** | 0.01 | - | 0.01 | - | - | - | 0.00 | - | - | - | **0.02** | |
|  | **AN** | 0.02 | - | 0.01 | - | - | - | 0.01 | - | 0.04 | - | **0.09** | 0.00 | - | 0.00 | - | - | - | 0.01 | - | - | - | **0.01** | |
|  | **MI** | 0.01 | - | 0.04 | - | - | - | - | - | 0.02 | - | **0.08** | 0.01 | - | 0.03 | - | - | - | 0.00 | - | - | - | **0.04** | |
|  | **AI** | 0.01 | - | 0.01 | - | - | - | 0.02 | - | 0.03 | - | **0.08** | 0.01 | - | 0.01 | - | - | - | 0.01 | - | - | - | **0.02** | |
|  | **AA** | 0.01 | - | 0.02 | - | - | - | 0.01 | - | 0.01 | - | **0.05** | 0.00 | - | 0.01 | - | - | - | 0.01 | - | - | - | **0.02** | |
| Children's park | **SB** | 0.02 | - | 0.02 | - | - | - | 0.01 | - | 0.03 | - | **0.08** | 0.01 | - | 0.01 | - | - | - | 0.00 | - | - | - | **0.02** | |
|  | **PB** | 0.03 | - | 0.02 | - | - | - | 0.01 | - | 0.08 | - | **0.14** | 0.00 | - | 0.01 | - | - | - | 0.01 | - | - | - | **0.02** | |
|  | **TX** | 0.03 | - | 0.01 | - | - | - | 0.01 | - | 0.02 | - | **0.07** | 0.01 | - | 0.01 | - | - | - | 0.01 | - | - | - | **0.02** | |
|  | **OT** | 0.04 | - | 0.02 | - | - | - | - | - | 0.03 | - | **0.10** | 0.02 | - | 0.01 | - | - | - | 0.01 | - | - | - | **0.04** | |
|  | **LO** | 0.02 | - | 0.02 | - | - | - | 0.01 | - | 0.03 | - | **0.08** | 0.01 | - | 0.01 | - | - | - | 0.01 | - | - | - | **0.03** | |
|  | **LH** | 0.02 | - | 0.01 | - | - | - | - | - | 0.02 | - | **0.05** | 0.02 | - | 0.01 | - | - | - | 0.00 | - | - | - | **0.03** | |
|  | **AR** | 0.01 | - | 0.01 | - | - | - | - | - | 0.01 | - | **0.03** | 0.01 | - | 0.00 | - | - | - | 0.00 | - | - | - | **0.02** | |
|  | **PU** | 0.02 | - | 0.01 | - | - | - | 0.01 | - | 0.05 | - | **0.09** | 0.00 | - | 0.01 | - | - | - | 0.01 | - | - | - | **0.02** | |
