# Supplementary material for: Simplified vs extended in vitro methods for the evaluation of bioaccessibility of metals and metalloids present in urban recreational soils
Source: Environ Sci Pollut Res Int. 2025 Feb 9;32(9):5358–70. doi: 10.1007/s11356-025-36017-y (PMC11868185; doi:10.1007/s11356-025-36017-y)
Supplement: Supplementary file 8 — (DOCX 17.3 KB) [file 11356_2025_36017_MOESM8_ESM.docx]

|  |  | CR children, SBET | | | | CR children, RIVM | | | |
| --- | --- | --- | --- | --- | --- | --- | --- | --- | --- |
|  |  | Cr | As | Pb | **Total CR** | Cr | As | Pb | **Total CR** |
| Urban park | MU | 4.59E-08 | 1.59E-06 | 1.12E-06 | **2.75E-06** | - | 2.15E-06 | 6.64E-09 | **2.16E-06** |
|  | BE | 7.77E-08 | 3.26E-06 | 9.76E-07 | **4.31E-06** | - | 3.08E-06 | 4.29E-09 | **3.09E-06** |
|  | LV | 4.03E-08 | 2.21E-06 | 1.32E-07 | **2.38E-06** | - | 1.10E-06 | 0.00E+00 | **1.10E-06** |
|  | AM | 4.79E-08 | 2.06E-06 | 6.55E-07 | **2.77E-06** | - | 2.36E-06 | 2.98E-09 | **2.36E-06** |
|  | SI | 1.21E-07 | 4.02E-06 | 1.92E-06 | **6.06E-06** | - | 2.80E-06 | 1.91E-08 | **2.82E-06** |
|  | MA | 2.49E-08 | 2.72E-06 | 5.52E-07 | **3.30E-06** | - | 3.28E-06 | 8.06E-09 | **3.29E-06** |
|  | SA | 1.12E-07 | 1.21E-06 | 1.54E-07 | **1.48E-06** | - | 4.05E-07 | 0.00E+00 | **4.05E-07** |
|  | EG | 1.37E-08 | 2.58E-06 | 3.52E-07 | **2.94E-06** | - | 1.92E-06 | 0.00E+00 | **1.92E-06** |
|  | CE | 2.69E-08 | 1.32E-06 | 6.16E-07 | **1.96E-06** | - | 2.91E-06 | 1.07E-08 | **2.92E-06** |
|  | MP | 2.03E-07 | 3.35E-06 | 8.49E-07 | **4.40E-06** | - | 4.14E-06 | 2.69E-09 | **4.14E-06** |
|  | AE | 3.63E-07 | 2.90E-06 | 1.85E-06 | **5.12E-06** | - | 3.83E-06 | 2.93E-09 | **3.83E-06** |
|  | PG | 1.01E-07 | 4.10E-06 | 1.83E-06 | **6.03E-06** | - | 3.89E-06 | 5.34E-09 | **3.90E-06** |
|  | HE | 1.60E-07 | 3.26E-06 | 1.04E-06 | **4.45E-06** | - | 1.24E-06 | 0.00E+00 | **1.24E-06** |
|  | UM | 1.06E-07 | 1.69E-06 | 6.09E-07 | **2.40E-06** | - | 2.13E-06 | 0.00E+00 | **2.13E-06** |
|  | AN | 5.01E-07 | 5.32E-06 | 1.22E-06 | **7.04E-06** | - | 2.50E-06 | 0.00E+00 | **2.50E-06** |
|  | MI | 4.78E-08 | 8.65E-07 | 6.44E-07 | **1.56E-06** | - | 1.07E-06 | 3.64E-09 | **1.08E-06** |
|  | AI | 5.11E-08 | 7.64E-06 | 1.00E-06 | **8.69E-06** | - | 3.58E-06 | 6.13E-09 | **3.59E-06** |
|  | SB | 3.92E-08 | 2.33E-06 | 9.04E-07 | **3.27E-06** | - | 1.75E-06 | 4.64E-09 | **1.75E-06** |
| Children's park | PB | 7.84E-08 | 4.45E-06 | 2.47E-06 | **7.00E-06** | - | 3.55E-06 | 1.72E-08 | **3.57E-06** |
|  | TX | 4.39E-08 | 3.61E-06 | 6.74E-07 | **4.33E-06** | - | 3.24E-06 | 0.00E+00 | **3.24E-06** |
|  | OT | 1.71E-07 | 1.98E-06 | 9.28E-07 | **3.08E-06** | - | 2.52E-06 | 5.76E-09 | **2.52E-06** |
|  | LO | 8.69E-08 | 4.84E-06 | 7.72E-07 | **5.70E-06** | - | 3.10E-06 | 1.60E-08 | **3.12E-06** |
|  | LH | 3.50E-08 | 1.39E-06 | 4.93E-07 | **1.92E-06** | - | 1.80E-06 | 1.04E-08 | **1.81E-06** |
|  | AR | 1.70E-08 | 1.92E-06 | 3.67E-07 | **2.31E-06** | - | 2.09E-06 | 3.34E-08 | **2.12E-06** |
|  | PU | 5.38E-08 | 5.17E-06 | 2.99E-06 | **8.22E-06** | - | 3.60E-06 | 2.58E-09 | **3.60E-06** |
|  | AA | 3.11E-08 | 2.93E-06 | 2.42E-07 | **3.21E-06** | - | 2.54E-06 | 0.00E+00 | **2.54E-06** |

**Supplementary Table 8**. Cr index (for children) of Cr, As and Pb in the studied 26 urban soils and Total CR index of each soil (calculated as the sum of the three CR values).
