# Supplementary material for: Simplified vs extended in vitro methods for the evaluation of bioaccessibility of metals and metalloids present in urban recreational soils
Source: Environ Sci Pollut Res Int. 2025 Feb 9;32(9):5358–70. doi: 10.1007/s11356-025-36017-y (PMC11868185; doi:10.1007/s11356-025-36017-y)
Supplement: Supplementary file 9 — (DOCX 17.8 KB) [file 11356_2025_36017_MOESM9_ESM.docx]

|  |  | CR adults, SBET | | | | CR adults, RIVM | | | |
| --- | --- | --- | --- | --- | --- | --- | --- | --- | --- |
|  |  | Cr | As | Pb | **Total CR** | Cr | As | Pb | **Total CR** |
| Urban park | MU | 1.97E-08 | 6.80E-07 | 4.78E-07 | **1.18E-06** | - | 9.21E-07 | 2.84E-09 | **9.24E-07** |
|  | BE | 3.33E-08 | 1.40E-06 | 4.18E-07 | **1.85E-06** | - | 1.32E-06 | 1.84E-09 | **1.32E-06** |
|  | LV | 1.73E-08 | 9.46E-07 | 5.64E-08 | **1.02E-06** | - | 4.71E-07 | 0.00E+00 | **4.71E-07** |
|  | AM | 2.05E-08 | 8.85E-07 | 2.81E-07 | **1.19E-06** | - | 1.01E-06 | 1.28E-09 | **1.01E-06** |
|  | SI | 5.20E-08 | 1.72E-06 | 8.21E-07 | **2.60E-06** | - | 1.20E-06 | 8.20E-09 | **1.21E-06** |
|  | MA | 1.07E-08 | 1.17E-06 | 2.36E-07 | **1.41E-06** | - | 1.41E-06 | 3.45E-09 | **1.41E-06** |
|  | SA | 4.79E-08 | 5.20E-07 | 6.60E-08 | **6.34E-07** | - | 1.74E-07 | 0.00E+00 | **1.74E-07** |
|  | EG | 5.87E-09 | 1.10E-06 | 1.51E-07 | **1.26E-06** | - | 8.23E-07 | 0.00E+00 | **8.23E-07** |
|  | CE | 1.15E-08 | 5.64E-07 | 2.64E-07 | **8.40E-07** | - | 1.25E-06 | 4.59E-09 | **1.25E-06** |
|  | MP | 8.68E-08 | 1.44E-06 | 3.64E-07 | **1.89E-06** | - | 1.77E-06 | 1.15E-09 | **1.78E-06** |
|  | AE | 1.56E-07 | 1.24E-06 | 7.93E-07 | **2.19E-06** | - | 1.64E-06 | 1.26E-09 | **1.64E-06** |
|  | PG | 4.31E-08 | 1.76E-06 | 7.84E-07 | **2.59E-06** | - | 1.67E-06 | 2.29E-09 | **1.67E-06** |
|  | HE | 6.88E-08 | 1.40E-06 | 4.44E-07 | **1.91E-06** | - | 5.32E-07 | 0.00E+00 | **5.32E-07** |
|  | UM | 4.55E-08 | 7.24E-07 | 2.61E-07 | **1.03E-06** | - | 9.11E-07 | 0.00E+00 | **9.11E-07** |
|  | AN | 2.15E-07 | 2.28E-06 | 5.21E-07 | **3.02E-06** | - | 1.07E-06 | 0.00E+00 | **1.07E-06** |
|  | MI | 2.05E-08 | 3.71E-07 | 2.76E-07 | **6.67E-07** | - | 4.59E-07 | 1.56E-09 | **4.61E-07** |
|  | AI | 2.19E-08 | 3.27E-06 | 4.30E-07 | **3.72E-06** | - | 1.54E-06 | 2.63E-09 | **1.54E-06** |
|  | SB | 1.68E-08 | 9.98E-07 | 3.88E-07 | **1.40E-06** | - | 7.49E-07 | 1.99E-09 | **7.51E-07** |
| Children's park | PB | 3.36E-08 | 1.91E-06 | 1.06E-06 | **3.00E-06** | - | 1.52E-06 | 7.38E-09 | **1.53E-06** |
|  | TX | 1.88E-08 | 1.55E-06 | 2.89E-07 | **1.85E-06** | - | 1.39E-06 | 0.00E+00 | **1.39E-06** |
|  | OT | 7.33E-08 | 8.50E-07 | 3.98E-07 | **1.32E-06** | - | 1.08E-06 | 2.47E-09 | **1.08E-06** |
|  | LO | 3.72E-08 | 2.07E-06 | 3.31E-07 | **2.44E-06** | - | 1.33E-06 | 6.86E-09 | **1.34E-06** |
|  | LH | 1.50E-08 | 5.95E-07 | 2.11E-07 | **8.21E-07** | - | 7.72E-07 | 4.48E-09 | **7.76E-07** |
|  | AR | 7.27E-09 | 8.25E-07 | 1.57E-07 | **9.90E-07** | - | 8.94E-07 | 1.43E-08 | **9.09E-07** |
|  | PU | 2.31E-08 | 2.22E-06 | 1.28E-06 | **3.52E-06** | - | 1.54E-06 | 1.11E-09 | **1.54E-06** |
|  | AA | 1.33E-08 | 1.26E-06 | 1.04E-07 | **1.37E-06** | - | 1.09E-06 | 0.00E+00 | **1.09E-06** |

**Supplementary Table 8**. Cr index (for adults) of Cr, As and Pb in the studied 26 urban soils and Total CR index of each soil (calculated as the sum of the three CR values).
